# Network models of primary melanoma microenvironments identify key melanoma regulators underlying prognosis

**DOI:** 10.1038/s41467-021-21457-0

**Published:** 2021-02-22

**Authors:** Won-Min Song, Praveen Agrawal, Richard Von Itter, Barbara Fontanals-Cirera, Minghui Wang, Xianxiao Zhou, Lara K. Mahal, Eva Hernando, Bin Zhang

**Affiliations:** 1 Department of Genetics and Genomic Sciences, Icahn School of Medicine at Mount Sinai, One Gustave L. Levy Place, New York, NY USA; 2 Mount Sinai Center for Transformative Disease Modeling, Icahn School of Medicine at Mount Sinai, One Gustave L. Levy Place, New York, NY USA; 3 Icahn Institute for Data Science and Genomic Technology, Icahn School of Medicine at Mount Sinai, One Gustave L. Levy Place, New York, NY USA; 4 Department of Pathology, NYU Grossman School of Medicine, New York, NY USA; 5 Interdisciplinary Melanoma Cooperative Group, Perlmutter Cancer Center at NYU Langone Health, New York, NY USA; 6 Department of Chemistry, University of Alberta, Edmonton, Canada; 7 Department of Pharmacological Sciences, Icahn School of Medicine at Mount Sinai, One Gustave L. Levy Place, New York, NY USA

**Keywords:** Cancer, Computational biology and bioinformatics, Genetics, Immune evasion, CD4-positive T cells

## Abstract

Melanoma is the most lethal skin malignancy, driven by genetic and epigenetic alterations in the complex tumour microenvironment. While large-scale molecular profiling of melanoma has identified molecular signatures associated with melanoma progression, comprehensive systems-level modeling remains elusive. This study builds up predictive gene network models of molecular alterations in primary melanoma by integrating large-scale bulk-based multi-omic and single-cell transcriptomic data. Incorporating clinical, epigenetic, and proteomic data into these networks reveals key subnetworks, cell types, and regulators underlying melanoma progression. Tumors with high immune infiltrates are found to be associated with good prognosis, presumably due to induced CD8+ T-cell cytotoxicity, via *MYO1F*-mediated M1-polarization of macrophages. Seventeen key drivers of the gene subnetworks associated with poor prognosis, including the transcription factor *ZNF180*, are tested for their pro-tumorigenic effects in vitro. The anti-tumor effect of silencing *ZNF180* is further validated using in vivo xenografts. Experimentally validated targets of *ZNF180* are enriched in the *ZNF180* centered network and the known pathways such as melanoma cell maintenance and immune cell infiltration. The transcriptional networks and their critical regulators provide insights into the molecular mechanisms of melanomagenesis and pave the way for developing therapeutic strategies for melanoma.

## Abstract (web summary)

While the molecular profiling of melanoma progression has been extensively characterised by large-scale studies, there is still need for the comprehensive integration of such datasets. Here the authors construct predictive gene network models for prognostic and therapeutic purposes.

## Introduction

Melanoma is a highly aggressive tumor that accounts for less than 5% of all skin cancers, but 80% of skin cancer-related deaths^1^. Melanoma is a malignancy of pigment-producing cells, i.e., melanocytes, located predominantly in the skin. These pigments play protective roles against ultraviolet (UV) radiation in healthy individuals, safeguarding cells against DNA damage. Accumulation of UV exposure in melanocytes contributes to high mutational burden^1,2^, conferring mitogenic signals via defective DNA repair and replicative mechanisms and inducing oncogenic mutations such as those observed in *BRAF* and *NRAS*^3^. Mutations in *BRAF*, *NRAS*, *NF1*, and *KIT* have been all implicated as melanoma drivers^1,2,4,5^.

Primary melanomas are heterogeneous in terms of molecular and clinical features, as well as their tumor microenvironment. Interaction with diverse stromal cells can activate pro-invasive programs such as an epithelial-to-mesenchymal transition in some primary tumor cells, leading to metastatic dissemination^6,7^. Dynamic changes in the tumor microenvironment can also lead to immune surveillance escape^8,9^ and are predictive of melanoma prognosis^10^. Within the tumor microenvironment, tumor-reactive T lymphocytes play a central role in suppressing tumor growth by infiltrating into malignant lesions and selectively killing cancer cells^11,12^. However, some subclones of melanoma tumors evade the immunosurveillance by intra-tumoral expression of programmed cell death ligand 1 (PD-L1), which binds to the co-inhibitory checkpoint receptor, programmed cell death protein 1 (PD-1)^13,14^.

While understanding the heterogeneity is critical for patient treatment, several factors have hampered a comprehensive molecular characterization of primary melanoma tumors. First, most large-scale, multi-omics studies focus on metastatic tumors or combine analysis of primary and metastatic tumors^2^. Further, these large-scale studies are often bulk-based and confounded by the diversity of cell-types within cancer. This complicates the identification of cell type-specific signaling circuits within the microenvironment. Although machine learning methods such as CIBERSORT^15^ and ESTIMATE^16^ can estimate relative cell compositions in bulk samples to some degree, they cannot replace the high-resolution analysis of cell-type-specific interactions from scRNA-seq.

Systems biology, especially network biology approaches, have proven effective for integrating diverse, large-scale datasets in complex human diseases^17–39^. Here, we applied an integrative multi-scale gene network analysis framework to jointly analyze the primary melanoma bulk RNA-sequencing data from The Cancer Genome Atlas (denote as pSKCM) and a published single-cell transcriptomic dataset^40^. We hypothesized that co-expressed gene modules associated with the patients’ prognosis capture dysregulated pathways in primary melanoma etiology. By generating prognosis gene signatures from the TCGA data, we were able to intersect these signatures with gene modules and identify the enriched modules, subnetworks, and network drivers as pro-tumorigenic regulators of primary melanoma. Similarly, gene signatures associated with (epi-)genomic alterations were utilized to inform gene modules affected by these alterations. This integrative approach has proven effective in identifying causal molecular alterations in complex diseases such as Alzheimer’s disease^41,42^, asthma^43^, breast cancer^44^, and gastric cancer^45^.

Our study revealed key immune cell types and signaling pathways, and predicted their regulators underlying primary tumors with varying degrees of tumor infiltration by jointly analyzing bulk and single-cell data. Key findings were replicated in independent bulk^46^ and scRNA-seq datasets^40,47^. Further, the predicted pro-tumorigenic regulators of melanoma were validated via *siRNA* screening in vitro and in vivo xenografts.

## Results

### Integrative network biology analysis of primary melanoma

**Fig. 1:**
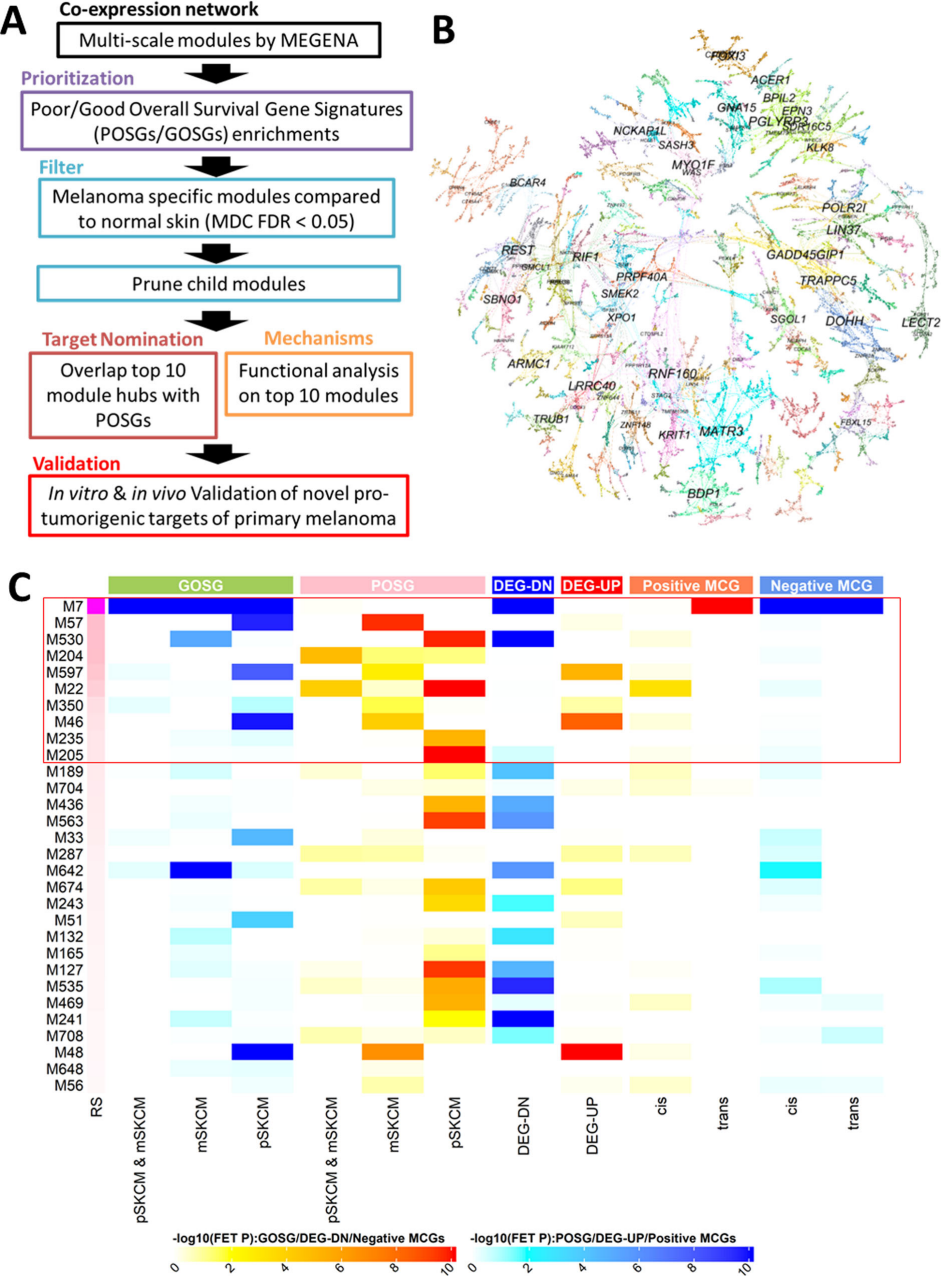
Analytic flow of the co-expression network analysis of the primary skin cutaneous melanoma cohort in TCGA. **A** Overall workflow. **B** Global co-expression network of the primary melanoma samples from TCGA. Gene modules identified with the default resolution parameter *α* = 1 are shown in distinct colors. Highly connected hub genes are labeled with respective gene symbols. **C** Heatmap representation of molecular features associated with the top 30 gene modules by survival analysis. Heatmap color represents an enrichment of respective gene signatures in the modules. GOSG represents the good prognosis associated genes in pSKCM and/or metastatic melanoma (mSKCM) cohorts. POSG includes the poor prognosis associated genes in pSKCM and/or mSKCM cohorts. DEG-UP and DEG-DN are the upregulated and downregulated gene signatures in tumors in comparison with adjacent normal tissues, respectively. MCG represents the methylation correlated genes (MCG). MCG with a positive (negative) correlation between the gene and a CpG site is called Positive (negative) MCG. MCG is also classified into *cis*- or *trans*-regulation dependent on the distance between the respective CpG site’s location and the genomic location of the gene (see the “Methods”).

We constructed a gene co-expression network from the bulk-based primary skin cutaneous melanoma (pSKCM) RNA-seq data in The Cancer Genome Atlas (TCGA) to identify co-expressed gene modules (subnetworks) and their key regulators by multiscale embedded gene co-expression network analysis (MEGENA) (Fig. 1, Supplemental Fig. 2A, B)^48^. A total of 221 gene modules were prioritized by enrichment for the genes associated with overall survival and known primary melanoma-specific pathways (see “Methods”).

Among the top ten ranked modules, five (M7, M597, M57, M46, and M350) were enriched for the genes whose upregulation was associated with increased overall survival (denoted GOSG; Supplementary Fig. 2B) while the others (M530, M22, M205, M204, and M235; Supplemental Fig. 2A) were enriched for the genes whose upregulation was associated with poor prognosis (denoted POSG). In general, not many POSG enriched modules are associated with known pathways or functions. Only one POSG enriched module M22 significantly overlaps with generic transcription pathways curated by REACTOME (corrected Fisher’s Exact Test *p* value (cFET *p*) = 3.45E − 54, 13 fold-enrichment(FE)), due to co-expressions of zinc finger proteins.

In contrast, the top-ranked GOSG enriched module, M7 (Fig. 1C; cFET *p* = 5.76E − 193, 13.2 FE) is characterized by immune response pathways, including the hallmark IFN-γ response (cFET *p* = 4.95E − 95, 9.29 FE), the *CTLA4* pathway (cFET *p* = 6.20E − 8, 10.6 FE), TNFα signaling via NFκB (cFET *p* = 8.36E − 16, 3.6 FE) and the T-cell receptor alpha pathway (cFET *p* = 2.56E − 11, 13.1 FE). Hyper-methylation in cis-CpG sites of several genes in M7 was associated with their downregulation (cis-MCG: cFET *p* = 4.01E − 10, 2.43 FE). As altered gene expression and methylation affect each other directly and indirectly, we dissected the causal relationships between these genes and the altered CpG sites. We identified *PTPN6* and *PTPRCAP* as potential modulators of epigenetic regulation of the leukocyte activation pathway in M7 (see Epigenetic silencing of T-cell activation leads to poor prognosis in Supplementary Methods; epigenetic regulatory network in Supplementary Data 6).

### Upregulated immune response subnetwork associated with good overall survival

The module most strongly associated with a good prognosis, M7, is comprised of sub-modules^48^ reflecting distinct immune response pathways, including leukocyte activation (M110) and antigen presentation (M112, Supplementary Fig. 3B). M112 captured the PD1/PD-L1 signaling pathway, including PD-L1 and several key regulators of the PD-L1 transcription pathway as its hubs, such as *STAT1* and *IRF1*^49^. The immune regulatory role of M112 was further supported by its enrichment of the genes upregulated by PD1 ligation in T cells^50^ (cFET *p* = 1.62E − 21, 16.6 FE). The hubs of M7 were also associated with the T-cell activation pathway (cFET *p* = 3.86E − 10, 40.2 FE; Supplementary Data 3).

We dissected the immune cell populations involved in M7 and its sub-modules by integrating the scRNA-seq data comprised of 4645 cells from 19 melanoma patients (GEO accession: GSE72056)^40^. The individual cells were annotated with the published and inferred cell types, including melanoma, T cells (CD3+), macrophages, endothelial, and cancer-associated fibroblasts. We further classified T-cell subpopulations into CD8+ cells, CD4+ T cells, resting memory (RM) CD4+ T cells, and M1-polarized macrophages (M1 macrophages) (Supplementary Fig. 4A; see “Methods”). Genes in M7 sub-modules were expressed in distinct cell types, predominantly melanoma cells (expressed in M401, M402, M110, M639, and M642), M1-macrophages (expressed in M400, M401, and M642), B-cells (expressed in M111), CD8+ T cells (expressed in M399, M401, M402, and M403) and CD4+ T cells (expressed in M399, M401, and M402, Supplementary Fig. 4C).

**Fig. 2:**
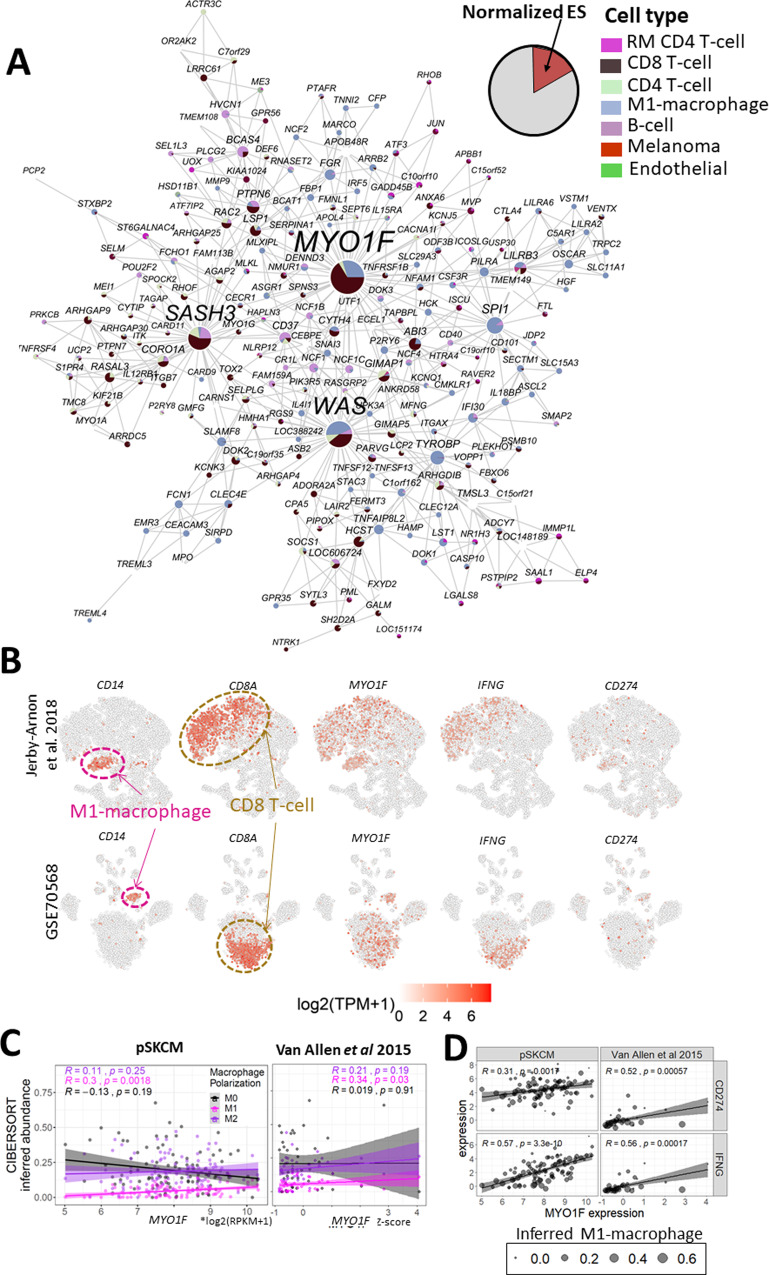
MYO1F as a potential regulator of IFN-γ response in primary melanoma microenvironment. **A** The network of a POSG-enriched module, M401. Pie size in a node piechart is proportional to the specificity of the respective gene in a cell type, defined as an enrichment score, −log10(FET FDR), which is derived from the enrichment test for the cells expressing the respective gene and the cells that are predicted to belong to the cell type. Enrichment scores for each gene are normalized by the sum of the enrichment scores for all the seven cell types. Cell types with FET FDR < 0.05 are shown in the piecharts. Pie color indicates different cell types: resting memory (RM) CD4 T cell (magenta), CD8 T cell (dark brown), CD4 T cell (palegreen), M1-macrophage (skyblue), B-cell (dark plum), Melanoma (dark red), Endothelial (green). **B** *MYO1F* is expressed in M1-macrophages and CD8 T cells along with PD-L1 (CD274). tSNE plots of the scRNA-seq datasets from GSE72056 and Jerby-Arnon et al.^47^ show the cellwise expressions of macrophage marker (*CD14*) and CD8 T-cell marker (*CD8A*) along with *MYO1F*, INF-γ (*IFNG*), and PD-L1 (*CD274*). The scale for expression levels by log2(TPM + 1) values are shown at the bottom. **C** Scatter plots between *MYO1F* and CIBERSORT inferred macrophage populations (M0–M2) in the pSKCM and Van Allen et al.^46^ cohorts. 95% confidence intervals for Spearman correlations between *MYO1F* and macrophages are (−0.29, 0.33) for M0, (0.036, 0.59) for M1 and (−0.11, 0.49) for M2. **D** Spearman correlation between *MYO1F* expression and PD-L1(*CD274*)/INF-γ (*IFNG*) in the pSKCM and Van Allen et al. 2015 cohorts. Point size is proportional to inferred M1-macrophage abundance. The 95% confidence intervals of the correlation coefficients for *CD274* and *IFNG* in pSKCM are (0.12, 0.47) and (0.42, 0.69), respectively. The confidence intervals for *CD274* and *IFNG* in the Van Allen et al. 2015 cohort are (0.25, 0.72) and (0.30, 0.74), respectively.

To understand gene interactions within each cell population, we constructed a co-expression network for each cell cluster (labeled as CLS1, CLS2, etc.), termed as sc-networks (see Supplementary Fig. 4B). Then, we evaluated the concordance between the pSKCM network and the sc-networks to identify cell populations involved in the bulk-based gene interactions. For example, the interferon-γ (IFN-γ) response modules M401 and M402 significantly overlap the macrophage-enriched modules in the CLS3 sc-network (cFET *p* = 9.48E − 20). This makes sense as IFN-γ is a known activator of macrophages^51^. The *MYO1F* neighborhood from the pSKCM network was significantly enriched in the *MYO1F* neighborhood in the CLS3 network (Fig. 2A; cFET = 2.12E − 6, 211 FE; Supplementary Fig. 4G). In single-cell transcriptomes, *MYO1F* was consistently expressed in M1-macrophages and IFN-γ-secreting CD8+ T cells in the discovery cohort and an independent cohort of 33 melanoma tumors^47^ (Fig. 2B; Supplementary Fig. 5B; Supplementary Fig. 6). These *MYO1F*^high^ M1-macrophages cells expressed PD-L1 (*CD274* in Fig. 2B), and *MYO1F*^high^ CD8+ T cells showed high IFN-γ expression (*IFNG* in Fig. 2B). Given that M1-macrophages are pro-inflammatory and interact with cytotoxic CD8+ T cells^52^, these results suggest *MYO1F* may play an essential role in IFN-γ/PD-L1 signaling in primary melanoma.

The findings from single-cell transcriptomes are consistent with those from the pSKCM bulk RNA-seq data. *MYO1F* was correlated with the inferred M1-macrophage abundance in pSKCM but not with M0-/M2-macrophages (Fig. 2C). We leveraged the published immune subtypes of the pSKCM samples by Thorsson et al.^53^ to clarify which immune subtype is associated with *MYO1F*. *MYO1F* showed predominantly high expression in the IFN-γ dominant subtype, C2 (Supplementary Fig. 7A), and was correlated with inferred M1/M2 macrophage ratio and CD8+ T-cell abundance within C2 (Supplementary Fig. 7B).

In agreement with these observations, *MYO1F* has been identified as an upstream regulator that hyper-activates macrophage *STAT1*, leading to M1-polarization and IFN-γ secretion by stimulating intercellular adhesion^54^. *MYO1F* may also regulate leukocytes. *MYO1F* is associated with the T cell co-inhibitory receptor, PD-L1, in both bulk cohorts (Fig. 2D). PD-L1 upstream genes such as *IRF1* and *STAT1* are more highly expressed in *MYO1F*^high^ M1-macrophages than in the other CD19+ monocyte/macrophages (Supplementary Fig. 6).

To validate the IFN-γ response subnetwork in macrophages captured in the pSKCM network, we curated the loss-of-function (LoF) signatures of a key IFN-γ response pathway regulator, *STAT1*, from bone marrow-derived macrophages (BMM) of *Stat1* knockout (*Stat1*-KO) mice (GSE48970) by querying the CREED database^55^. As expected, the downregulated genes from *Stat1*-KO BMM are significantly enriched in the *STAT1* centered subnetwork in the pSKCM network (Supplementary Fig. 3C; cFET *p* = 2.80E − 31, 34.6 FE). Furthermore, *STAT1* expression was correlated with inferred M1-macrophage abundance by CIBERSORT in pSKCM (Spearman *ρ* = 0.59, *p* = 4.28E − 11; Supplementary Fig. 3E). These results support the *STAT1* centered network depicts IFN-γ response of macrophages in pSKCM. Altogether, *MYO1F* may modulate IFN-γ response in M1-macrophages and cytotoxic CD8+ T cells via the *STAT1*/*IRF1*/*PD-L1* axis.

### Upregulation of intra-tumoral DNA repair and mRNA splicing pathways associates with poor prognosis

We further characterized the top-ranked modules associated with poor prognosis, including M530, M22, M205, M204, and M235 (Supplementary Fig. 2A). Although these modules are not associated with known pathways and functions in the MSigDB, the hub genes in these modules include known regulators of pre-mRNA splicing such as serine-arginine protein-like factors, *SFRS3*, and *SFRS13A*^56^, which are the hubs of M235, and a spliceosome associated protein, *SR140* as a hub of M530^57^. M205 includes a nuclear pore component (*NUP205*)^58^ and a splicing factor (*TNPO3*)^59^. On the other hand, the hubs of M22 include DNA repair regulators such as *FANCM*^60^ (a component of the Fanconi anemia core complex) and *ZNF180*, which has a locus associated with aberrant genomic rearrangements^61^. Interestingly, *FANCM* and *ZNF180* gene expressions were correlated with expression of a DNA mismatch repair protein, MSH2, from the reverse-phase protein array (RPPA) datasets (Supplementary Data 9) with *ρ* = 0.530 (adjusted *p* = 4.53E − 2) and 0.529 (adjusted *p* = 4.48E − 2), respectively. In melanoma, high expression of DNA repair genes including *MSH2* is associated with metastasis and may explain their resistance to chemo- and radio-therapies through maintaining the genetic stability^62^. These data suggest DNA repair and mRNA splicing pathways are linked to poor prognosis in primary melanoma.

**Fig. 3:**
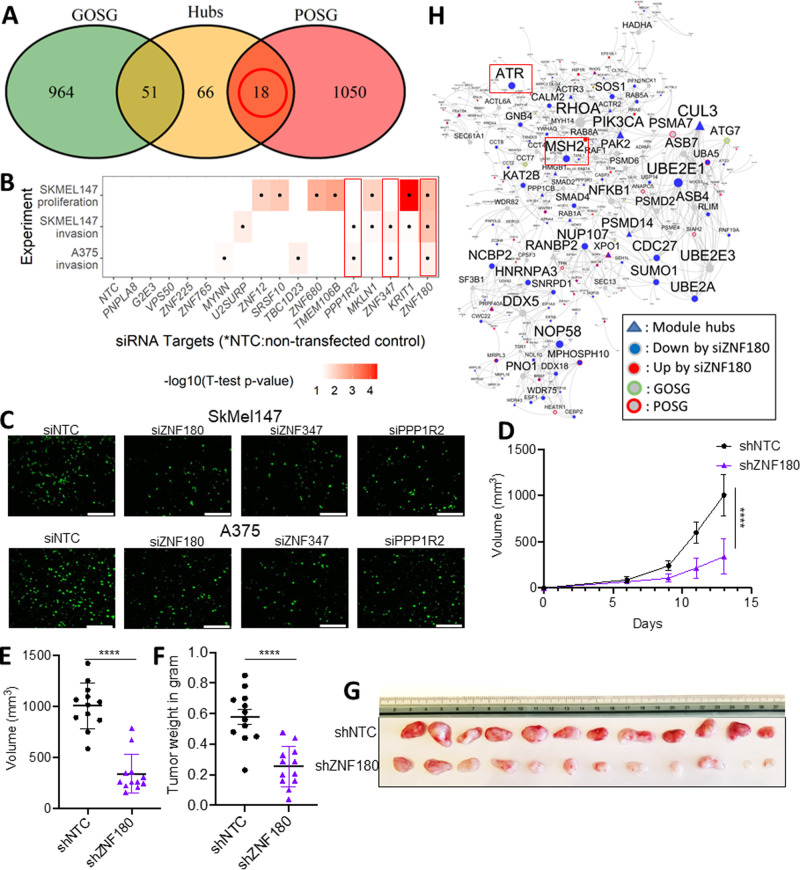
Systematic identification and validation of *ZNF180* as a promising target in primary melanoma. **A** Intersections between the poor survival gene signature from pSKCM (pSKCM-POSG), and the good survival gene signature (pSKCM-GOSG), and the hubs of the top ten modules. Eighteen hub genes which are also pSKCM-POSG were nominated for siRNA screenings. **B** siRNA screening of the nominated targets for proliferation in (top) and invasion (middle) in SKmel147, and invasion in A375 cells (bottom). The heatmap shows −log10(*t* test *p* value) to remark the significance of the siRNA screening results, and significant results are marked by dots. Red rectangles highlight consistently validated genes (*ZNF180*, *ZNF347* and *PPP1R2*). **C** Trans-well matrigel invasion assays on SKmel147 and A375 cells transduced with siNTC or siZNF180, siZNF347, and siPPP1R2. Invading cells were quantified by counting the number of SKmel147 and A375 cells that invaded into the basal side of Matrigel-coated trans-well inserts after 8 and 6 h respectively, *n* = 5 fields per replicate; 3 replicates per condition, representative images are shown. Scale bar corresponds to 100 µm. **D** Growth curve representing average tumor volume over time in mice injected with shNTC or shZNF180-transduced SKmel147 cells (*n* = 12 per group). Tumor volume (**E**) and tumor mass (**F**), and (**G**) images of resected tumors taken 13 days post injection. Data are presented as mean ± SD in (**D**–**F**). **** denotes significance of the comparison by two-tailed unpaired *t* test by *p* < 0.0001. **H** M25 captures protein interactions curated in the STRING database with a confidence score >70%. Node colors and border colors denote differentially expressed genes by siZNF180 (siZNF180-DEG) and pSKCM survival signatures as shown in the legend.

To identify primary melanoma prognosis regulators, we examined the intersection between the previously identified top ten modules’ hub genes and the poor prognosis signatures (Fig. 3A). This scheme nominated 18 regulators, which were more likely to be essential for melanoma cell viability than the poor prognosis signature alone (Wilcox *p* value = 3.03E − 4; Supplementary Fig. 10) based on CRISPRi screening data in the Achilles database^63^. These potential regulators’ gene expression profiles were correlated with the inferred tumor purity score by ESTIMATE, supporting them as tumor cell-intrinsic genes (Supplementary Fig. 9). Higher expression of these key regulators in tumors was associated with lower CD8+ T cell as inferred by CIBERSORT (Supplementary Fig. 9), suggesting intra-tumoral expression of these genes was associated with low infiltration of cytotoxic T cells. Therefore, these predicted regulator genes capture intra-tumoral pathways essential to tumor viability and confer poor prognosis in primary melanoma.

### Systematic validation of candidate network drivers of poor prognosis

To validate the 18 candidate regulators of the molecular networks underlying primary melanoma prognosis, we performed siRNA knockdown experiments in SKmel147 (*NRAS* mutant) and A375 (*BRAF* mutant) cell lines (see siRNA screening of candidate targets in Supplementary Methods). Of the 18 candidates, siRNAs for 17 were readily available for high-throughput screening assays to assess the impact on cell growth and invasion. siRNA-mediated knockdown of five genes (*KRIT1*, *ZNF680*, *SRSF10*, *ZNF180*, and *TMEM160B*) in SKmel147 cells significantly reduced cell growth (*p* value < 0.05), relative to non-targeting controls (NTC) (top, Fig. 3B). Knockdown of eight key regulators (*PPP1R2*, *G2E3*, *MKLN1*, *ZNF225*, *ZNF180*, *ZNF347*, *KRIT1*, and *U2SURP* (*SR140*)) in SKmel147 cells significantly reduced invasion (middle, Fig. 3B). A similar siRNA invasion screening on A375 cells yielded significant differences for five genes: *TBC1D23*, *PPP1R2*, *ZNF180*, *MYNN*, and *ZNF347* (bottom, Fig. 3B). Altogether, we validated the pro-tumorigenic effects of 13 (76.4%) of 17 candidate regulators tested in SKmel147 and A375 cells (Table 1).

**Table 1:** List of top nominated key genes associated with prognosis in primary melanoma tumors.

Hub gene	Connectivity	Cox *P*	log-rank *P*	Module
*PPP1R2*^a,b^	17	0.048877	0.04945458	M530
TBC1D23^b^	17	0.05934	0.04945458	M530
MYNN^b^	16	0.002709	0.00286385	M530
SR140 (U2SURP)^a^	16	0.002702	0.00142604	M530
G2E3^a^	25	0.018412	0.04492035	M22
ZNF180^c,a,b^	24	0.004064	0.00422203	M22
ZNF225^a^	23	0.206873	0.02719113	M22
ZNF765	19	0.002705	0.01459658	M22
ZNF347^a,b^	17	0.013813	0.01459658	M22
SFRS13A (SRSF10)^a^	25	0.00736	0.00562611	M235
KRIT1^a,c^	47	0.017451	0.00616876	M205
TMEM106B^c^	33	0.016207	0.00063893	M205
PNPLA8	25	0.034177	0.00142604	M205
CCDC132 (VPS50)	22	0.18037	0.021213	M205
ZNF680^c^	20	0.02294	0.00142604	M205
ZNF12	18	0.001513	0.00286385	M205
MKLN1^a^	18	0.082066	0.03678236	M205

^a^Validated genes from invasion screening in SKmel147 cells.

^b^Validated genes from invasion screening in A375 cells.

^c^Validated genes from proliferation screening in SKmel147 cells.

Connectivity of a node (i.e., a gene) is the number of links incident to the node in the co-expression network of TCGA-pSKCM. Cox P (i.e., Cox *p* value) was obtained from a two-sided Cox proportional hazard model for the respective gene expressions. Log-rank *p* (i.e. log-rank *p* value) was obtained by grouping patients by median expressions of the respective genes via a two-sided log-rank test. The module shows the gene module membership of the respective genes. Genes highlighted by bold fonts were chosen for comprehensive transcriptome analysis by RNA-sequencing of SKmel147 cells transfected with the respective siRNAs.

### *ZNF180* silencing antagonizes melanoma cell proliferation and invasion in vitro and in vivo

Among the 13 successfully validated functional candidate genes, *PPP1R2* (a hub of M530)*, ZNF180*, and *ZNF347* (hubs of M22) showed robust effects in multiple siRNA functional assays. Transient depletion of *PPP1R2*, *ZNF180*, and *ZNF347* significantly reduced the invasive capacity of SKmel147 and A375 cells in trans-well invasion assays (middle, Fig. 3B). Of these candidates, only the knock-down of ZNF180 showed significant anti-proliferative effects in SKmel147.

Therefore, we further investigated the anti-proliferative effect of *ZNF180* silencing in vivo. Lower *ZNF180* transcript and protein levels confirmed effective knock-down in shZNF180–SKmel147 cells relative to shNTC-transduced control cells (Supplementary Fig. 14). SKmel147 cells stably infected with control (shNTC) or shZNF180 expressing lentivirus were injected into the flanks of NOD/Shi-scid/IL-2Rgamma null (NSG) mice (*n* = 12/group). Mice injected with shZNF180 transduced cells showed significantly reduced tumor growth (*p* < 0.0001) and weight at termination (*p* < 0.0001) compared to mice injected with cells carrying a non-targeting shRNA (Fig. 3D–G). This supports the role of *ZNF180* in melanoma growth in vivo. Together, our data indicate that *ZNF180* is essential for melanoma growth both in vitro and in vivo.

RNA-sequencing revealed transcriptional changes elicited by *ZNF180* silencing in SKmel147 cells (see RNA-sequencing in Supplementary Methods). Genes differentially expressed in response to silencing *ZNF180* (termed siZNF180-DEGs; Supplementary Data 8) were significantly enriched in the subnetwork of *ZNF180* in the pSKCM network using threshold-free rank–rank hypergeometric overlap test (see Analysis of RNA-sequencing data of siRNA knock-down SKmel147 cells in Supplementary Methods; Supplementary Fig. 11E). Moreover, the genes downregulated by siZNF180 were enriched in the four-layer network neighborhood of *ZNF180* in the pSKCM network (Supplementary Fig. 12A; cFET *p* = 1.41E − 83, 3.69 FE), thus validating the topological structure of pSKCM gene network. Such findings were replicated in the co-expression network analyses of the metastasis SKCM (mSKCM) and Van Allen et al.^46^ cohorts (Supplementary Fig. 12B).

### *ZNF180* is a multifunctional driver of primary melanoma etiology

The siZNF180-DEGs were associated with several aberrant signaling pathways in cancer and pSKCM prognosis. The downregulated genes (siZNF180-DN) significantly overlap with pSKCM-POSG (cFET *p* = 1.07E − 172, 3.00 FE). The intersection between these two signatures was associated with the cell cycle pathway (cFET *p* = 2.15E − 6, 3.60 FE). On the contrary, the upregulated genes (siZNF180-UP) significantly overlapped the good prognosis signatures in pSKCM (FET FDR = 7.32E − 54, 2.03 FE), and their intersection was associated with the generic immune system pathway (cFET *p* = 6.37E − 3, 2.64 FE).

We further examined a potential correlation between *ZNF180* and protein expressions from the RPPA to identify candidate interacting proteins. Protein expressions of mismatch repair protein, MSH2, and plasminogen activator inhibitor, PAI-1, were significantly correlated with *ZNF180* expression with *ρ* = 0.54 (adjusted *p* = 4.48E − 2) and −0.44 (adjusted *p* = 4.83E − 2), respectively. Particularly, MSH2 closely interacted with the genes down-regulated by siZNF180 in the protein-protein interaction network (Fig. 3H). Given the role of MSH2 and DNA repair in melanoma cells to maintain tumor cells’ genetic stability, the data suggests *ZNF180* may play a role in regulating these pro-tumorigenic pathways.

**Fig. 4:**
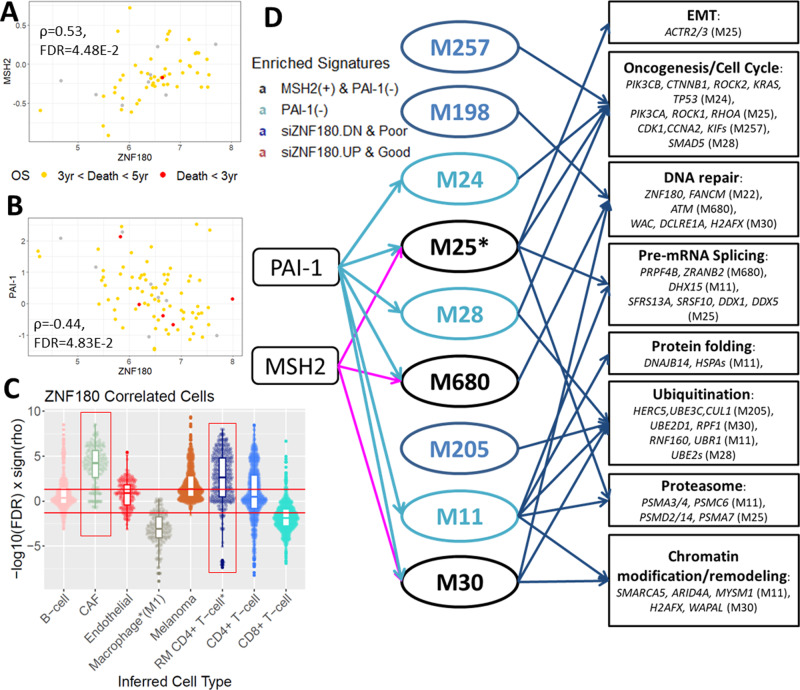
Dissection of *ZNF180*-regulated pathways in melanoma. **A**, **B** Correlations between *ZNF180* expressions (*x*-axis) and protein expressions (*y*-axis; **A**: MSH2, **B**: PAI-1) by RPPA. Dot color indicates patients’ prognosis in overall survival (death within 3 years of follow-up: red, otherwise yellow). **C** Cell abundance is correlated with *ZNF180* expressions in pSKCM. The relative abundance of 4645 cells with annotated cell types (in *x*-axis) were inferred across 103 pSKCM samples by cell population mapping (CPM) algorithm in the scBio package. The inferred abundance was then tested for correlation with *ZNF180* expressions in pSKCM. Each dot represents the correlation between the relative abundance of one cell from the scRNA-seq with *ZNF180* expressions in pSKCM. the *x*-axis is cell types of the respective cells, and the *y*-axis is −log10(FDR) signed by Spearman’s correlation coefficient. The red horizontal lines mark FDR = 0.05 threshold, and red rectangles highlight the most distinctively correlated cell types. The border of a boxplot show the lower quantile, median, or upper quantiles while a whisker spans towards the minima and the maxima. **D** Summary of the pathways associated with the modules potentially regulated by *ZNF180*. Enriched signatures in the modules are color-coded.

The gene modules enriched for the siZNF180-DEGs more comprehensively captured the signaling pathways controlled by *ZNF180*. The modules enriched in the genes upregulated by siZNF180 included tumor suppressors and negative cell cycle regulators (M597; Supplementary Table 1). In contrast, those enriched for the genes downregulated by siZNF180 were associated with DNA repair, epithelial–mesenchymal transition (M25), oncogenesis (M24, M25, and M257), pre-mRNA splicing (M25 and M30), protein modification/degradation and chromatin modification/remodeling (Fig. 4D; see Differentially expressed pathways by *ZNF180* suppression in SKMEL147 cells in vitro in Supplementary Methods).

Notably, M25 emerged as a core module regulated by *ZNF180* as it was enriched for not only the down-regulated genes by siZNF180 but also the poor prognosis signatures from pSKCM (Fig. 4D). M25 was associated with oncogenic signaling pathways, including MYC targets (Hallmark MYC targets V1: FET FDR = 9.43E − 4, 2.58 FE). M25 also constituted a coherent protein interaction network, comprised of high confidence interactions in the STRING database (>70% confidence; Fig. 3D)^64^. The protein interaction network within M25 included established oncogenes such as *ROCK1*, *PIK3CA, RHOA*^65,66^, and the key DNA repair regulators, *MSH2*, and *ATR* (Fig. 3D). These results indicated close interactions between the DNA repair signaling and oncogenic signaling pathways in primary melanoma.

To understand the cell populations regulated by *ZNF180*, we deconvoluted the pSKCM bulk samples into de novo cell types from the melanoma single-cell transcriptome studies using the cellular population mapping (CPM) algorithm implemented in “*scBio*” R package^67^. *ZNF180* expression was consistently correlated with increased cancer-associated fibroblasts and decreased M1-macrophage abundance in two studies, GSE72056^40^ (Fig. 4C), and Jerby-Arnon et al.^47^ (Supplementary Fig. 5D). Overall, our results show that *ZNF180* is a multifunctional driver of oncogenic and DNA repair pathways that may also modulate tumor infiltration by M1 macrophages and cancer-associated fibroblasts.

## Discussion

This study constructed a compendium of multi-scale gene networks from bulk and single-cell RNA-seq data to dissect complex molecular interactions and co-regulation underlying primary melanoma pathogenesis. Our analysis revealed a number of pathways and regulators underlying primary melanoma and its microenvironment. The upregulation of the immune response module, M7, is a strong positive prognostic factor and correlated with increases in CD8+ T cells and M1-macrophages. In contrast, the regulators of the subnetworks predictive of poor prognosis highlighted intra-tumoral DNA repair pathways. We systematically tested the anti-tumoral effects of 17 candidates via siRNA knockdown experiments in vitro. *ZNF180* had the most consistent anti-tumor effects and was further validated in vivo.

Network drivers of key cancer modules as identified by MEGENA are potential regulators of many other genes, while networks provide functional context for how those regulators operate. While the traditional prognosis analysis offers a list of prognostic genes to identify enriched known pathways, such simple analyses are constrained on pre-collected knowledge-bases, limiting the discovery of de novo disease pathways. On the contrary, network-based findings offer mechanistic insights such as gene modules (i.e., de novo pathways) and hubs (i.e., potential regulators) that complement traditional approaches. Identification of *MYO1F* and *ZNF180* and their intrinsic network models supports the advantages of the integrative network approach adopted in this study.

Notably, *MYO1F* emerged as a potential regulator in macrophages. *MYO1F* is an influential hub in the bulk-based pSKCM network (i.e., a top hub in the M401 module) and the network from the pro-inflammatory M1 macrophage cell cluster in the single-cell transcriptomic data (i.e., a hub in M1-macrophage enriched cell cluster). We confirmed high *MYO1F* expression in M1 macrophage populations in the melanoma single-cell transcriptomic data (Fig. 2B).

The data indicate *MYO1F* plays a role in the INF-γ pathway, mediated by *STAT1* activation in M1-macrophages. *MYO1F* regulates M1-polarization in macrophages within intestinal mucosa by stimulating intercellular adhesion and promotes IFN-γ secretion via hyper-activating *STAT1*, a hub gene of the same immune response module, M76. High IFN-γ secretion in this pathway is consistent with the significant upregulation of *MYO1F* in an immune-subtype in the INF-γ dominant microenvironment by Thorsson et al.^53^. To establish the role of *STAT1* in this pathway within melanoma, we developed a *STAT1*-centered network model from pSKCM, and intersected it with *STAT1* perturbation signature in the *Stat1*-KO mouse model from bone-marrow-derived macrophages (BMM). We observed a significant overlap between the *STAT1*-centered network and *STAT1* perturbation signature from BMM (Supplementary Fig. 3C). *STAT1* expression was correlated with inferred M1-macrophage abundance by CIBERSORT in pSKCM (Spearman *ρ* = 0.59, *p* = 4.28E − 11; Supplementary Fig. 3E). As there is only one tumor with somatic *STAT1* mutation in pSKCM, we do not have the power to determine the relationship between *STAT1* mutation and M1 macrophage abundance. Thus, the *STAT1*-centered network presents the detailed gene interactions in the *STAT1* pathways in macrophages. Given the close interaction between *STAT1* and *MYO1F* (Pearson *ρ* = 0.32, *p* = 9.88E − 4), these observations also support the regulatory roles of *MYO1F* in M1-polarization and *STAT1* activation in macrophages, leading to increased IFN-γ secretion in the melanoma microenvironment.

The data also suggest that *MYO1F* may play a role in mediating interactions between CD8+ T cells and M1-macrophages underlying activated adaptive immune systems in the primary melanoma microenvironment. *MYO1F* mRNA expression was significantly correlated with IFN-γ (i.e., *IFNG*) in an independent melanoma bulk transcriptomic dataset (Fig. 2D). In the single-cell transcriptomic data, IFN-γ was expressed in CD8+ T cells, while *MYO1F* was expressed in CD8+ T cells and M1-macrophages (Fig. 2B). Indeed, co-localization of M1-macrophages and CD8+ T cells alone can induce IFN-γ production by CD8+ T cells^68^, enhancing their motility and cytotoxicity^69^. Taken together with *MYO1F*’s regulatory role in driving M1-polarization, the correlation between IFN-γ and *MYO1F* in the bulk RNA-seq datasets may reflect such enhanced cytotoxicity due to *MYO1F*-mediated M1-polarization, which in turn increases the abundance of co-localized M1-macrophages and CD8+ T cells.

High expression of a co-inhibitory T-cell checkpoint, PD-L1, was also found to be associated with a good prognosis in pSKCM (log-rank *p* = 1.32E − 2). Although an increase in PD-L1 was implicated in cytotoxic T-cell inhibition and thereby promoted immune-surveillance escape in solid tumors^49,70^, the prognostic relevance of high PD-L1 expression has been somehow controversial. In a broad spectrum of solid tumors, several studies reported high PD-L1 was associated with worse outcomes^70–72^, whereas high PD-L1 in metastatic melanoma was found to be correlated with higher infiltrating T-cell content and better prognosis^73^. In our study, high PD-L1 may reflect the abundance of *MYO1F*^high^ M1-macrophages modulating an INF-γ dominant microenvironment and is independent of T-cell suppression. We observed high PD-L1 expression mostly within the *MYO1F*^high^ M1-macrophages, indicating they were predominant sources of PD-L1 (Fig. 2B). Interestingly, the correlation between *CD274* (i.e., PD-L1 expressions) and *IFNG* (i.e., INF-γ expressions) (Fig. 2D) also suggests high PD-L1 as a marker for the INF-γ dominant microenvironment.

**Fig. 5:**
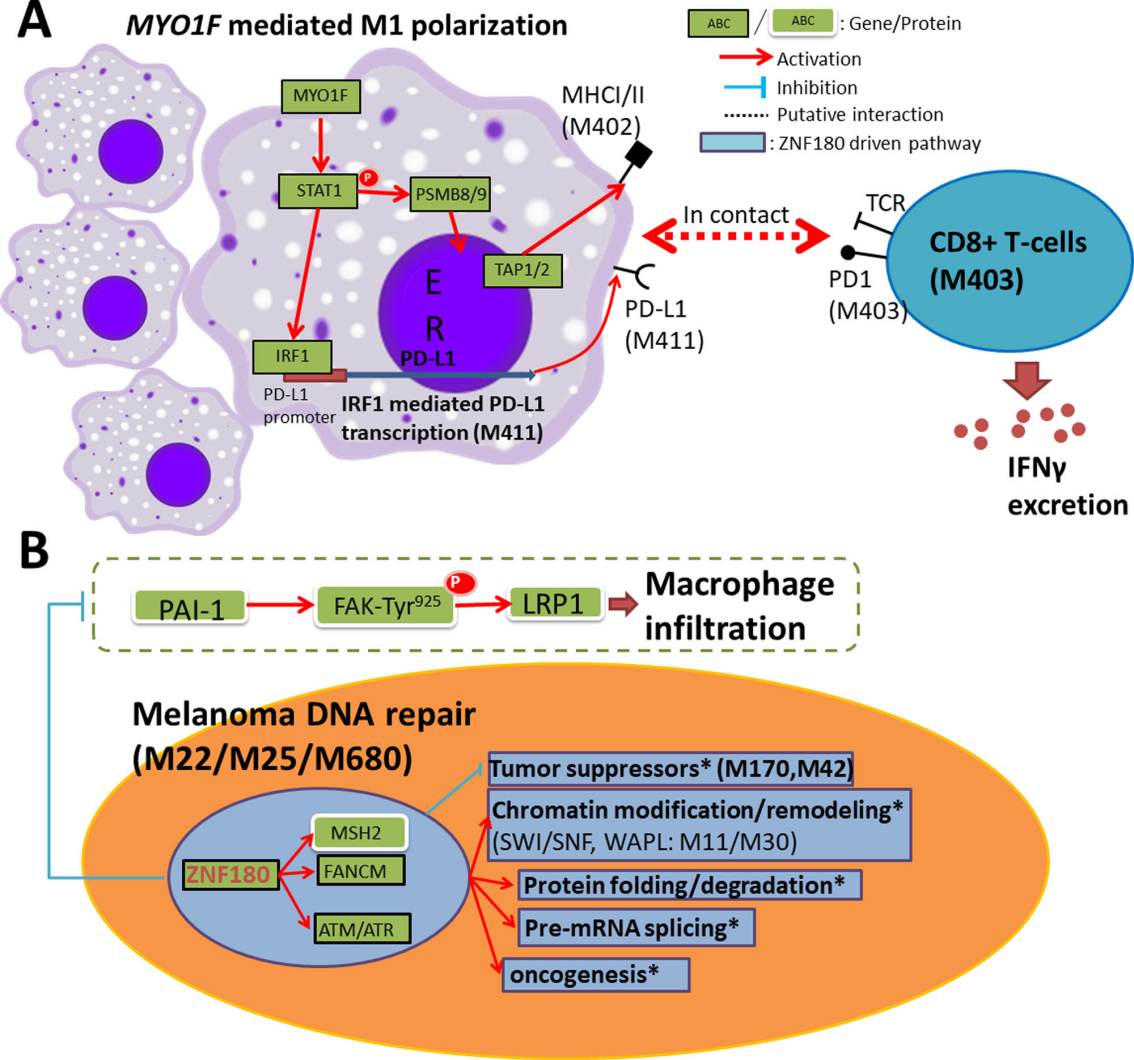
Key signaling pathways in primary melanoma captured by the bulk pSKCM and single-cell gene networks. **A** *MYO1F* mediated M1-polarization of macrophages is associated with a good prognosis. Contact with CD8+ T cells leads to increased IFNγ excretion in the tumor microenvironment. **B** *ZNF180* overexpression drives DNA repair and downstream melanoma pathways and suppresses macrophage infiltration via decreased PAI-1 protein expression.

Together, the *MYO1F-PD-L1*/*PD1-IFNγ* axis was identified as a prognostic pathway involving overall IFN-γ production and antitumoral activities of cytotoxic T lymphocytes (Fig. 5A).

Among 17 nominated targets from the pSKCM network, *ZNF180* showed the most consistent pro-tumorigenic effect (Fig. 4D). Consistent with the suppression of DNA repair pathways in melanoma cells by the knockdown of *ZNF180*, the module primarily regulated by siZNF180, M25, harbors interactions between DNA repair pathways involving MSH2, and oncogenic pathways involving *PIK3CA*, Rho kinases, and GTPases. Indeed, overexpression of MSH2 in primary melanoma was associated with poor prognosis, likely by enabling tumor cells to proliferate and metastasize as reported^62^. In contrast, several modules upregulated by silencing of *ZNF180* include tumor suppressors such as *BRMS1* (Breast cancer metastasis suppressor 1; within M170)^74^ and negative regulator of cell cycle *GADD45GIP1*^75^ as hub genes. In pSKCM, PAI-1 protein expression is negatively correlated with *ZNF180* expression. PAI-1 has been shown to promote macrophage infiltration in melanoma via phosphorylation of FAK-Tyr^925^ ^76^ (Fig. 5B), suggesting *ZNF180* may also regulate immune cell infiltration in primary melanoma.

In conclusion, this study has identified multi-facetted axes of tumor progression and immune evasion of primary melanoma including enhanced CD8+ T-cell cytotoxicity likely via *MYO1F* mediated macrophage M1-polarization and *ZNF180* as a molecular regulator of DNA repair and immune cell infiltration in melanoma cells. The network models have revealed additional regulators that have not been fully characterized in this study. More mechanistic studies (e.g., genetic and epigenetic regulation) using immune-competent models are needed to understand the functional impact of the prioritized regulators of melanoma (e.g., *MYO1F* and *ZNF180*) on tumor progression and immune modulation.

## Methods

### Bioinformatics methods

#### Gene expression data processing

Ilumina HiSeq RNA Sequencing data, processed by reads per kilobase per million (RPKM) method from TCGA (i.e. RNASeqV2), has been downloaded and comprehensive data quality control has been performed. Primary and metastatic tumor samples were collectively processed by log2(RPKM + 1) transform, followed by quantile-normalization. From then, we split the data into primary-/metastatic-specific gene expression matrix, corrected for batch effects by the center, platform, and tissue source site (transcription starting site (TSS)) ids from TCGA sample barcodes, and corrected for confounding factors including race, age, and gender by capturing residuals with intercepts from linear regression model by lm() function from R software (version 3.4.2). This resulted in 103/353 annotated primary/metastatic tumor tissue samples across 19,047 genes.

#### Correlation analysis between methylation and gene expression

We firstly processed the level 3 DNA methylation data from The Cancer Genome Atlas (TCGA), corresponding to *M*-values from Illumina Infinium Human DNA Methylation 450k platform. Upon quantile-normalization, the M-values were further adjusted for batch variables, including TSS, plate and center ids, and gender and age at diagnosis by taking residuals with intercepts from the linear model, gene expression ~α_1_TSS + α_2_plate + α_3_center id + α_4_gender + α_5_age + α_0_. We identified gene expressions associated with DNA methylation within the metastatic tumor or primary tumor samples by pairwise Spearman’s correlation analysis. *Cis*-regulated genes were defined by applying 1.5 kbps from TSS.

#### Extraction of prognostic gene signatures

We performed a Wald test to systematically evaluate the prognostic significance of stratifying patients by individual gene expressions by the medians. We used the function survdiff() from the survival 2.41-3R package^77^ to calculate log-rank *p* value for overall survival outcome within primary and metastatic tumors separately and applied log-rank *p* value < 0.05. The common prognostic gene signature across primary and metastatic tumors was then defined as the intersection of the respective significant gene signatures and was utilized for ranking the gene modules through enrichment test.

#### Co-expression network analysis

Gene co-expression networks were constructed by multiscale embedded gene co-expression network analysis (MEGENA)^48,78,79^. MEGENA first selects gene pairs with significant correlations (FDR < 0.05). The significant gene pairs are then sorted by absolute Pearson’s correlation coefficients and then embedded sequentially by running down the sorted gene pairs to test if each pair can be drawn on the three-dimensional topological sphere without crossing other edges (i.e., planarity test). This edge embedding process is terminated if any of the following three conditions are met: (1) no more edges can be added, (2) the number of embedded edges reaches the maximal number of edges for the planar network, i.e., 3(|*V*| − 2) (|*V*| = the number of genes), and (3) the significant list of gene pairs has been tested fully for planarity^80^. The resulting co-expression network belongs to a class of geometrical networks called “planar filtered networks (PFNs)” which can be drawn on the surface of the sphere without any link intersections^80^.

PFN then goes through unsupervised clustering to identify network clusters (i.e., gene modules) at various compactness resolutions by multi-scale clustering analysis (MCA). MCA splits the parent module into child modules by searching for a partition optimizing for Newman’s modularity (*Q*)^81^. Then, the compactness of each child module is evaluated by the compactness measure *υ*, defined as below

1 $$\upsilon = \frac{{\overline {\rm{SPD}} }}{{\log (\left| V \right|)^\alpha }}$$

where *V* is the set of genes in the network, $\overline {\rm{SPD}}$ is the average of shortest path distances of all node pairs, and *α* is the resolution parameter. Given that the denominator $\log (\left| V \right|)^\alpha$ is the hallmark of the small-world property represented by the scaling relation $\widehat {\rm{SPD}}\sim \log (|V|)$ when *α* = 1, *υ* measures the coherence of a network’s topology^82^. Therefore, a smaller *α* identifies more compact clusters. For a given cluster (network), MCA searches through a range of *α* values for a resolution scale that leads to more compact clusters than the parent cluster. The resulting gene modules are organized in a hierarchy that represents a multiscale organization of gene modules with different degrees of compactness. The hierarchy captures a series of higher-order relationships (i.e., parent) modules possessing children modules residing within these parent modules. MEGENA identifies more compact children modules within the parent modules^48^.

Candidate key drivers of gene modules are further identified as the nodes with significantly (*p* < 0.05) higher network connectivity than the randomly permuted planar networks^48^. Gene modules are then annotated by the enriched MSigDB signatures and associated with outcomes through the enrichment test of the previously identified gene signatures from the survival and differential expression analyses.

#### Integrative network analyses of pSKCM cohort

To handle large-scale multi-faceted Omics data in primary skin cutaneous melanoma (pSKCM), we developed an integrative network analysis framework to identify and prioritize altered molecular subnetworks in primary melanoma etiology. The approach is anchored in constructing a multiscale co-expression network by MEGENA^48^.

We next generated a set of gene signatures reflecting genomic and epigenomic alterations (Supplementary Fig. 1, IV; co-expression network analysis in Supplementary Methods). The functional impact of epigenetic alterations was determined by gene signatures correlated with CpG sites annotated in the Illumina Infinium Human DNA Methylation 450k platform. If the significant correlation is observed between expressions of gene *x* and methylation at a proximal CpG site (less than 1.5 kbps within TSS) with Bonferroni corrected *p* < 0.05, the gene is called a *cis-*methylation-correlated gene (*cis*-MCG). If the CpG site is beyond 1.5 kbps from the TSS, the gene is called a *trans*-MCG (Supplementary Data 5). Enrichment of these signatures in a gene module reflects the impact of epigenetic alterations on the module.

From the bulk-based TCGA pSKCM data, MEGENA identified 221 gene modules with at least 50 genes, which were prioritized by enrichment for overall survival associated genes in pSKCM (log-rank *p* < 0.05; extraction of prognostic gene signatures in Supplementary Methods; see Supplementary Data 1 for the gene signatures).

We filtered out the co-expression network modules from MEGENA (see Supplementary Fig. 2A for the scheme) by comparing their connectivity to the normal skin cases from the genotype-tissue expression (GTEx) consortium^83^, before the module ranking process. We hypothesized network connectivity of normal skin driven modules resemble that of a normal skin co-expression network. We tested pSKCM module connectivity against normal skin co-expression network from the GTEx^83^ via module differential connectivity analysis^41^. Gene modules with significant gain or loss of overall gene-gene connectivity, compared to normal skin with an overall false-discovery rate (FDR) < 0.05 threshold were retained (see Supplementary Data 2B for the summaries of ranked modules).

Then, the importance of each gene module was determined by enrichment of the prognostic signatures. Specifically, we performed Fisher’s Exact Test to assess overrepresentation of the common prognostic gene signature and utilized *p* value to rank order the modules. Given the module hierarchy in the PFN, module ranking by enrichment test results in redundancy in the ranking due to the overlap between each parent module and its child modules. To handle this, we retained only the best-ranked module in each branch of the module hierarchy.

#### Analysis of melanoma single-cell RNA sequencing data

We downloaded the log2(TPM/10 + 1) gene expression matrix from Gene Expression Omnibus under the accession number, GSE72056^40^ (denoted GSE72056). GSE72056 served as a discovery single-cell dataset in combination with the discovery bulk RNA-seq cohort, pSKCM. This dataset was comprised of 1257 malignant cells, 2064 T cells, 525 B cells, 125 macrophages, 65 endothelial cells, 61 cancer associated-fibroblasts and 52 natural killer cells. We characterized these cells in both unsupervised and supervised manners to annotate cell subpopulations.

We utilized the *scran* (1.10.1) R package for the unsupervised clustering analysis of the single-cell RNA-seq data. The unsupervised clustering based on random walk community detection, namely the walk trap algorithm^84^, was applied by constructing the *k*-nearest neighbor (*k*NN) graph in the first ten PCs. The optimal value *k* was determined by detecting the elbow in the entropy curve for *k*NN *ϵ* [2,√*N*_c_] (*N*_c_ = number of cells). *k*NN = 19 emerged as the optimal result, leading to 15 cell clusters with sizes ranging from 77 cells to 488 cells. Then, each cell cluster’s markers were identified by contrasting each cell cluster with the rest using *limma* (3.36.1) R package with FDR < 0.05 and fold increase > 1.2.

Cell type inference was performed independently from the clustering results based on the cell type markers identified by Schelker et al. 2017^85^. A decision-tree algorithm implemented in R package *rpart* (4.1-13) was used to train classifiers which were then subsequently applied to the entire set of the cells. Cell type assignment for each cell was further refined by a known cell type most enriched in the *k*NN of the cell (see Supplementary Data 4A for cell types in the scRNA-seq data from GSE72056). Compared to the published cell types identified in GSE72056^40^, we achieved an accuracy of 84% and all the cell clusters had distinct cell types. For each cell cluster, we then constructed a cell-cluster specific co-expression network by MEGENA. Lowly expressed genes were filtered out if they were expressed in less than 20 cells and gene pairs with significant Spearman’s correlations (FDR < 0.05) were taken as an input for MEGENA^48^.

To validate the findings from GSE72056, we analyzed the scRNA-seq data of 33 melanoma tumors by Jerby-Arnon et al.^47^. We performed the same unsupervised clustering using the kNN approach, yielding 17 cell clusters (Supplementary Fig. 5A; see Supplementary Data 4B for cell cluster annotation). The cell type of each cell cluster was determined by the marker genes of the key cell types identified through GSE72056 (shown in Supplementary Fig. 5B). The marker genes include *PTPRC* (immune cell marker), *CD3E* (T-cell marker), *CD8A* (CD8+ T-cell marker), *CD19* (B-cell marker), *CD14* (monocyte/macrophage marker), *MITF* (melanoma cell marker), *VWF*, *CDH5* (endothelial cell marker), *COL1A1*, and *FAP* (cancer-associated fibroblasts, CAFs)^85^. In addition, we checked the chemokine markers of macrophage polarization, adapted from Rozer^86^ and Duluc et al.^87^. As shown in Supplementary Fig. 6, the cell cluster 17 has the most pronounced expression of M1-macrophage-specific chemokines, hence named as M1-macrophage in Supplementary Fig. 5A.

#### Identification of cell subpopulation specificity of a gene

For a given gene *i*, we first identified a list of cells expressing the gene with a threshold TPM_*i*_ > 0 and then tested how these cells were enriched for the cells in each inferred cell type (i.e., cell subpopulation) by FET. Multiple-testing corrected FET *p* value (i.e., FDR) < 0.05 was used as a threshold to designate a cell type (i.e., cell subpopulation) for the gene.

#### Cell composition analysis of pSKCM transcriptome data

The RPKM data of the pSKCM samples were taken as an input for the CIBERSORT web app at https://cibersort.stanford.edu/^15^ with 100 permutations to identify the relative abundance of immune cell populations. LM22 markers derived from purified distinct immune cell populations were used to infer the abundance. The results are provided in Supplementary Data 7A. We also performed ESTIMATE^16^ to infer more granular cell compositions including tumor cells, immune and stromal cells to complement CIBERSORT results based on L22 immune markers (provided in Supplementary Data 7B).

However, cell type characteristics in the tumor microenvironment are substantially different from the healthy microenvironment and is disease dependent^67,85^. In this case, leveraging disease matched scRNA-seq has shown improvements in cell type abundance inference^67,85^. To increase the confidence in the inferred results, we used cell type-specific expression from the scRNA-seq data to infer the relative abundance of individual single cells in the bulk pSKCM samples using the CPM algorithm implemented in the scBio R package^67^. As a result, mapping scores were computed between bulk samples and individual cells from the single-cell transcriptomic data (Supplementary Data 7D).

Detailed cell types of some CD3+ T cells and macrophages were not available in the scRNA-seq data. We performed a correlation analysis between the CPM inferred and CIBERSORT inferred abundances as LM22 markers included detailed cell type characteristics to address this issue. Supplementary Fig. 4D (upper panel) shows that CIBERSORT confirmed the detailed cell types correspond to M1-macrophage and RM CD4+ T cells. Indeed, the CD3+ T cells without unassigned T-cell subtypes showed prominent CD4 expression (Supplementary Fig. 4E), supporting that it was a CD4+ T-cell subtype. Henceforth, this cell population is denoted RM CD4+ T cell.

#### In-silico validation of gene perturbation signatures

SKmel147 cells transduced with siRNA against the top three target genes (namely, *ZNF180*, *ZNF347*, and *PPP1R2*) from the screening were selected for RNA-sequencing to identify respective knockdown gene signatures by differential expression analysis. We validated the network structure around a gene subject to a functional perturbation (for instance, gene knockdown or overexpression) by testing enrichment of the respective differentially expressed gene signatures in the network neighborhoods of the perturbed genes. Specifically, *l*-layer neighborhood of a gene, *g*, is defined as the set of genes whose shortest paths to *g* consists of *l* edges at most. The network structure is successfully validated if the BH FDR corrected FET *p* value < 0.05 within the *l*-layer neighborhood. A network structure with more than 10% of the total number of nodes in the global will not be tested as we are more interested in testing the local structure around a gene.

#### Identification of cell populations expressing specific genes

Per gene *i*, we first identified the list of cell types expressing the gene with threshold *TPM*_*i*_ > 0. Then, the list of expressing cells was tested for enrichments in the inferred cell types by Fisher’s Exact Test (FET). FET FDR < 0.05 was used as a threshold to designate a cell type as expressing the gene *i*.

#### Module conservation analysis between pSKCM and scRNA-seq

Each gene module in the PFN from the pSKCM was compared against the cell-cluster specific modules from the scRNA-seq data through FET. Two modules are conserved if the Bonferroni corrected FET *p* value is smaller than 0.05, the enrichment fold-change is greater than 2, and the overlap includes at least 30% of the genes in each module. Supplementary Data 4C includes detailed information about the module conservation analysis.

### Experimental methods

#### Western blotting

SkMel147 melanoma cells were lysed in ice-cold RIPA buffer supplemented with protease inhibitor cocktail (Roche), centrifuged at 14,000 rpm at 4 °C for 20 min and the supernatant was collected. Protein in the supernatant was estimated by DC protein assay (Bio-rad). Totally, 20 µg of protein were resolved by NuPage 4–12% Bis–Tris (Invitrogen) and transferred onto Immobilon-P polyvinylidene fluoride membranes (Millipore). Membranes were washed with distilled water followed by blocking with 5% nonfat dry milk in Tris-buffered saline supplemented with 0.05% Tween-20 (TBST) for 2 h at room temperature. Membranes were washed briefly with TBST then incubated in anti-ZNF180 (1:1000; Sigma SAB1306465), and anti-Tubulin (1:5000; Sigma, T9026) primary antibodies diluted in 5% nonfat dry milk in TBST (0.05% Tween-20) and incubated on a plate shaker for overnight at 4 °C. Membranes were washed for 3 times with TBST followed by incubation with horseradish peroxidase-conjugated anti-mouse or anti-rabbit secondary antibodies (1:5000; Sigma). Membranes were washed three times with TBST then signals were detected using Clarity Western ECL Blotting Substrate (BioRad) and imaged in LICOR Odyssey Fc imaging system.

#### Cell lines and cell culture

Cell line SKmel147 was obtained from Dr. Alan Houghton’s laboratory (MSKCC, New York). A375 was purchased from American Type Culture Collection. Cells were cultured in DMEM (Invitrogen) containing 10% (v/v) fetal bovine serum and 1% (v/v) penicillin/streptomycin. Cell lines were maintained in a 5% CO_2_ incubator at 37 °C. Cell lines were routinely tested to exclude mycoplasma contamination.

#### Reverse transfection of siRNA

Transfection conditions were optimized for SKmel147 and A375 melanoma cell lines using fluorescein-labeled oligos (Block-IT, Invitrogen). Liposomal transfection complexes with siRNA pools for each of the candidate genes and with NTC (siRNA ON-target plus SMARTpools, Dharmacon, 50 nM) were generated with Lipofectamine 2000 (Invitrogen), following the manufacturer’s recommendations. Media was changed after 6 hr incubation with liposomal complexes. Forty-eight hours after initiation of transfection, cells were used for RNA extraction, or for proliferation or invasion assays.

#### RNA extraction, reverse transcription, and qRT- PCR

Total RNA was extracted using miRNeasy QIAGEN mini kit according to the manufacturer’s protocol. Totally, 500 ng of RNA were reverse transcribed using TaqMan RT reagents (Applied Biosystems) with random hexamers following the manufacturer’s recommendations. cDNA was diluted with RNase and DNase free water prior to use in a quantitative real-time polymerase chain reaction (qRT-PCR). GAPDH was used as a housekeeping gene in qRT-PCR. Transcripts were quantified by ABI StepOne Real-Time PCR system (Applied Biosystems) using Power SYBR Green PCR MasterMix and following 2-step cycling parameters: holding stage, 10 min at 95 °C, and cycling stage 40 cycles of 95 °C for 15 s followed by 60 °C for 1 min followed by melting curve stage. The experiment was performed in three technical replicates.

#### Cell proliferation assays

Transfected cells were seeded at 3 × 10^3^ cells per well in 96-well plates. The following day (day 0) and every 24 h after (up to 3 days), cells were fixed in 0.1% glutaraldehyde and stored in phosphate-buffered saline (PBS) at 4 °C. Cells were then stained with 0.5% crystal violet, dried, and dissolved with 15% acetic acid. Optical density was read at 590 nm. For normalization and control purposes, cells transfected with NTC (siNTC) were present on each experiment.

#### In vitro invasion assay

Cell invasion was measured using 24-well Fluoroblok inserts (8 mm Becton Dickinson). Optimization was performed for SKmel147 and A375 cell lines to identify assay time length and Matrigel concentration. Briefly, siRNA transfected SKmel147 or A375 cells (40,000 cells per insert) were suspended in the serum-free medium over a Matrigel coating (Becton Dickinson), and a medium supplemented with 10% serum was used as a chemo-attractant. Cells that invaded after 6–8 h were stained in 4 μg/ml Calcein AM dye (ThermoFisher) for 1 h and counted in 5 different fields using a fluorescent microscope. For each independent experiment, three replicates per condition were run. The average of cell counts from three replicates per condition was used for plotting results. For control, cells transfected with NTC (siNTC) were present in each experiment. Cell counts for each well were normalized to the mean counts (of replicate wells) for the corresponding condition in the cell input plate to control for cell proliferation effects that may have occurred between initiation of transfection and assay seeding.

#### RNA-sequencing of siRNA transfected SKmel147 cells

Total RNA was extracted 72 h post transfection of siNTC, siZNF180, siZNF347, or siPPP1R2 in SKmel147 cell line in three replicates. RNA was extracted using miRNeasy QIAGEN mini kit. RNA was then processed with Ribo-Zero rRNA Removal Kit (Illumina) to remove rRNA, and further processed into sequencing libraries using Illumina TruSeq Stranded Total RNA Library Prep, following manufacturer’s protocol. All libraries were sequenced on Illumina HiSeq2500 ($150 M, 50 bp paired-end).

#### In vivo validation of protumorigenic effects of ZNF180 in SKMEL147 xenografts

SKmel147 cells were infected with doxycycline (DOX)-inducible shNTC or shZNF180 lentivirus and selected with 2 μg/ml puromycin for 48 h. Cells were induced with 2 μg/ml DOX for 3 days, trypsinized, washed with PBS, then suspended in sterile PBS at a concentration of 2 × 10^6^ cells per 150 µl, and maintained on ice until injection. Immediately before injection, cell aliquots were mixed with Matrigel (Becton Dickinson). Totally, 150 µl of cell/matrigel (1:1) suspensions were injected subcutaneously in the right flank of NOD/Shi-scid/IL-2Rgamma null (NSG, Jackson labs #005557) 20-weeks-old male mice (*n* = 12 per group). Mice were fed DOX-containing food (200 mg/kg weight). When primary tumors were palpable (6 days post injection), length (*l*) and width (*w*) were measured with calipers, 3 times weekly over a period of 13 days. Tumor volume was calculated using the formula (*l* × *w*^2^)/2. Tumor weight was measured at endpoints. Animal experiments were conducted in accordance with guidelines set forth by the Institutional Animal Care and Use Committee (IACUC) of NYU (protocol # S16-00051).

#### Viral production

Totally, 4 × 10^6^ HEK293T cells were seeded per 10 cm tissue culture plate and incubated overnight at 37 °C and 5% CO_2_. After seeding, HEK293T were co-transfected with PLKO-Tet-On-shNTC or PLKO-tet-on-shZNF180 (shRNA sequence: CCGGTGTCCTTGTTGTGCATCAAAGCTCGAGCTTTGATGCACAACAAGGACATTTTTTG) lentiviral expression constructs (12 μg), viral packaging plasmid (psPAX2, 8 μg), and viral envelope plasmid (pMD2.G, 4 μg) using Lipofectamine 2000 (Invitrogen) following manufacturer’s recommendations. Viral supernatant was collected and 0.45 mm filtered at 48 h post transfection and stored at 4 °C for short-term use (1–5 days) or −20 °C for long-term storage (5–30 days).

#### In vivo xenograft

SKmel147 cells were infected with DOX-inducible shNTC or shZNF180 lentivirus and selected with 2 μg/ml puromycin for 48 h. Cells were induced with 2 μg/ml DOX for 3 days, trypsinized, washed with PBS, then suspended in sterile PBS at a concentration of 2 × 10^6^ cells per 150 μl, and maintained on ice until injection. Immediately before injection, cell aliquots were mixed with Matrigel (Becton Dickinson). Totally, 150 μl of cell/matrigel (1:1) suspensions were injected subcutaneously in the right flank of NOD/Shi-scid/IL-2Rgamma null (NSG, Jackson labs #005557) 20-weeks-old male mice (*n* = 12 per group). Mice were fed DOX-containing food (200 mg/kg weight). When primary tumors were palpable (6 days post injection), length (*l*) and width (*w*) were measured with calipers, 3 times weekly over a period of 13 days. Tumor volume was calculated using the formula (*l* × *w*^2^)/2. Tumor weight was measured at endpoints.

### Reporting summary

Further information on research design is available in the Nature Research Reporting Summary linked to this article.

## Supplementary information

Supplementary Information

Supplementary Data 1

Supplementary Data 2

Supplementary Data 3

Supplementary Data 4

Supplementary Data 5

Supplementary Data 6

Supplementary Data 7

Supplementary Data 8

Supplementary Data 9

Reporting Summary

Description of Additional Supplementary Files

## Data Availability

The discovery bulk gene expression data, analyzed as primary melanoma (pSKCM), are available from The Cancer Genome Atlas (TCGA) data portal (https://portal.gdc.cancer.gov/). The discovery single-cell RNA sequencing data is available from Gene Expression Omnibus (GEO) under accession number GSE72056), and we downloaded the normalized TPM values (file name: GSE72056_melanoma_single_cell_revised_v2.txt.gz). The validation single-cell RNA sequencing data from Jerby-Arnon et al. (2018) was downloaded from https://singlecell.broadinstitute.org/single_cell/study/SCP109/melanoma-immunotherapy-resistance. The clinical and gene expression data (z-score transformed from log-transformed FPKM values) for the validation bulk cohort^46^ were downloaded from cbioportal website, https://www.cbioportal.org/study/summary?id=skcm_dfci_2015. The RNA-sequencing data from siRNA silencing of *ZNF180*, *ZNF347* and *PPP1R2* are available in GEO under accession number GSE161385. In addition, the processed data are available from the corresponding author upon reasonable request. Source data are provided with this paper.

## Source data

Source Data

## Code availability

MEGENA software (version 1.3.7) is publicly available as an R package in The Comprehensive R Archive Network (CRAN) (https://cran.r-project.org/web/packages/MEGENA/index.html). The development version of MEGENA is available from GitHub repository (https://github.com/songw01/MEGENA,10.5281/zenodo.4276493). We utilized ‘cit’ package for the Causal Inference Test (CIT) of methylation changes to the transcriptome. Additional codes for detailed implementation of MEGENA, CIT, in silico gene-gene interaction validation and other codes are available at https://github.com/songw01/melanoma_network_codes.
