# Supplementary material for: Network models of primary melanoma microenvironments identify key melanoma regulators underlying prognosis
Source: Nat Commun. 2021 Feb 22;12:1214. doi: 10.1038/s41467-021-21457-0 (PMC7900178; doi:10.1038/s41467-021-21457-0)
Supplement: Supplementary file 1 — Supplementary Information [file 41467_2021_21457_MOESM1_ESM.pdf]

# SUPPLEMENTARY INFORMATION

## TABLE OF CONTENTS

**Supplementary Methods** .......... 2
Differentially Expressed Genes (DEGs) between Primary and Metastatic Tumors .......... 2
Prognostic Gene Signatures of Primary and Metastatic Tumors .......... 2
Epigenetic silencing of T-cell activation leads to poor prognosis .......... 2
Analysis of RNA-sequencing data of siRNA knock-down SKmel147 cells .......... 3
Evaluation of Target nomination strategy by network connectivity .......... 4
Differentially expressed pathways by ZNF180 suppression in SKMEL147 cells in vitro .......... 6
**Supplementary Tables** .......... 6
**Supplementary Figures** .......... 9

# SUPPLEMENTARY METHODS

## *DIFFERENTIALLY EXPRESSED GENES (DEGS) BETWEEN PRIMARY AND METASTATIC TUMORS*

Comparison of 103 primary tumor samples against 353 metastatic tumor samples yielded 3,066 up-regulated (DEG-UP) and 3,933 down-regulated genes (DEG-DN) with FDR q-value < 0.05. We identified enriched pathways and functions curated by Molecular Signature Database (MSigDB), namely c2 (Biocarta, KEGG and Reactome), c5 (GO-Biological Processes, GO-Cellular Components), GO-Molecular Functions) and h (Hallmark) collections. [1, 2] Some of the top enriched pathways and established signatures include: Hallmark allograft rejection (FET p < 6.39E-31, 2.81-fold enrichment) by down-regulated genes, and ectoderm development (FET p < 6.13E-19, 3.73-fold enrichment) by up-regulated genes in primary tumor samples.

## *PROGNOSTIC GENE SIGNATURES OF PRIMARY AND METASTATIC TUMORS*

We derived survival gene signatures from Primary Skin Cutaneous Melanoma from TCGA (pSKCM) to identify functions and pathways associated to prognosis. pSKCM included overall survival follow-up data for 470 unique patients with median follow-up of 890 days, mean period of 1,596 days and 156 recorded deaths. Prognostic significance of each gene was evaluated by comparing overall survival curves between patients with low-expression below the median, and patients with high-expression above the median expression. Within each group of primary and metastatic samples, good/poor survival gene signatures were then defined by genes showing significantly higher/lower rate of observed deaths in the high-expression groups than the low-expression groups with logrank p-value < 0.05 [3].

This analysis yielded 1,068 and 1,015 genes whose up-regulation is associated with poor or good outcomes respectively from primary tumors, and 1,013 and 1,361 genes whose up-regulation is associated with poor or good outcomes respectively from metastatic tumors by logrank p-value < 0.05 and Cox p-value < 0.05 (these signatures are provided in Supplementary Material 2)**.** Among these gene signatures, 44 and 197 genes are commonly associated with poor or good outcomes in both of primary and metastatic tumors, respectively. These signatures were significantly enriched for important signaling pathways and functions including cell cycle (corrected Fisher's Exact Test p-value (cFET p) = 2.23E-7, 16-fold enrichment (FE)) for the common poor prognosis genes, and immune system (cFET p = 3.13E-12, 9.3-FE), defense response (cFET p = 3.15E-12, 7.5-FE) and T-cell activation (cFET p = 4.74E-10, 20-FE) for the shared good prognosis genes.

## *EPIGENETIC SILENCING OF T-CELL ACTIVATION LEADS TO POOR PROGNOSIS*

To identify potential epigenetic regulation of the modules predictive of melanoma prognosis, we tested enrichments of cis-/trans-methylation correlated genes (MCGs) in the modules. Putative causal regulators mediating epigenetic regulation in key pathways were further determined by the Causal Inference Test (CIT) framework[4] (Supplementary Material 7), leading to an integrative causal network regulated by methylation (mCIT).

We leveraged Causality Inference Test (CIT) framework[5] to identify methylation changes impacting gene expression changes in the downstream. Briefly, CIT evaluates the statistical significance of a causal

trio chain, L→G→T, where L is causal genomic variants/alterations, G is intermediate causal regulator, and T is a trait whose change is induced by the upstream regulators. For performing CIT on methylations, we considered two types of trios as follows: i) CpG→$G_{cis}^{i}$→$G_{trans}^{j}$, and ii) $G_{trans}^{j}$→CpG→$G_{cis}^{i}$, accounting for cis-/trans-regulations by methylations in gene expressions[4]. We curated these trios by collecting significant methylation-gene expression pairs by Bonferroni corrected p-value < 0.05 by Spearman's correlation within cis-/trans-pairs, defined by 1.5kbps window around Transcription Starting Site (TSS). Significant causal trio chains were identified by CIT p-value < 0.05.

Negative *cis*-MCGs (i.e. decreased expression upon higher methylation at cis-CpG sites) enriched for T-cell activation pathway(cFET p = 3.94E-05, 7.37 FE). The key drivers in mCIT include *PTPRCAP* and *PTPN6* (Supplementary Figure 8A), two key signaling proteins in T-/B-cell activation [6, 7]. Lower expressions of the two genes were predictive of poor survival in pSKCM (Supplementary Figure 8C; log-rank p of *PTPRCAP* at median cutoff = 1.46E-2 and the log-rank p of *PTPN6* at median cutoff = 9.34E-4).

Motivated by the prognostic significance of *PTPN6* and *PTPRCAP* in pSKCM, we further classified the pSKCM patients by methylation in their cis-CpG sites into low- and highly-methylated groups in T-cell Activation CpGs (TA-CpG Low/High) via unsupervised clustering (the bottom heatmap, Supplementary Figure 2C). TA-CpG clusters were correlated significantly with patient-wise enrichments of immune response module, M7 (Wilcox p-value between TA-CpG Low and TA-CpG High: 2.56E-6). This shows higher methylations at TA-CpGs corresponds to suppressed immune response overall.

TA-CpG clusters were also associated to altered tumor micro-environment in pSKCM. We leveraged the Cell Population Mapping (CPM) algorithm in *'scBio'* R package[8] to deconvolute cell compositions in pSKCM by melanoma derived cell populations, and tested for differentially abundant cells between TA-CpG Low and TA-CpG High samples by Wilcoxon rank sum test. M1-macrophages and CD8+ T-cells were the most decreased cell types in TA-CpG High, while RM CD4+ T-cells and melanoma cells showed marked increases (Supplementary Figure 8B). In comparison to other T-cell subpopulations, Resting Memory (RM) CD4+ T-cells from scRNA-seq showed substantially lower expressions of key checkpoint genes such as PD-1 (FDR=1.17E-2, 0.90-fold decrease) and CTLA4 (FDR=4.98E-73, 0.12-fold decrease), and lower expressions in overall T-cell receptor signaling pathway (cFET = 1.62E-13, 3.01 FE).

Interestingly, a key T lymphocyte co-inhibitory checkpoint, PD1/PD-L1 axis, showed markedly increased interaction with a significant correlation of PD1 and PD-L1 expressions in TA-CpG High, compared to no correlation within TA-CpG Low (Supplementary Figure 4D). PD1 and PD-L1 expressions were also significantly higher in TA-CpG Low, coupled with an increased M1-macrophage population compared to TA-CpG High (Supplementary Figure 4D).

Overall, these results suggest epigenetic suppression of T-cell activation pathway may be mediated by *PTPN6* and *PTPRCAP*, and is coupled with increased resting memory CD4+ T-cells with low checkpoint receptor expressions such as PD-1 and CTLA4.

### *Analysis of RNA-sequencing data of siRNA knock-down SKmel147 cells*

Three replicates per siRNA of *PPP1R2*, *ZNF180* and *ZNF347* (denoted siPPP1R2/siZNF347/siZNF180) with three non-transfected control SKmel147 cells (denoted NTC) were subject to RNA-sequencing. The reads were aligned using STAR 2-pass mapping to hg19 genome[9]. Gene expressions for coding

sequences were called by utilizing "*featureCounts()*" function from R package *Rsubread*[10] by counting mapped reads to exonic regions, and summarizing to "gene_id" as the meta feature (GTF.featureType = "exon",GTF.attrType = "gene_id"). Multiple mapping reads were also counted as fractionated across the mapped regions (countMultiMappingReads = TRUE, fraction = TRUE). The resulting count matrix was normalized by Transcripts Per Million (TPM), followed by log2-transformation[11].

We performed differential gene expression analysis by contrasting siRNA transfected cells against NTC per targeted gene. We first investigated transfection efficiency and its effects on the final differential expression signature by examining relative suppression of respective target gene expressions in siRNA-transfected cells (Supplementary Figure 11A-C). We confirmed that gene expressions of the siRNA target genes were significantly suppressed in comparison to NTC samples (Supplementary Figure 11A), and identified dependency of differentially expressed gene (DEG) signatures on the siRNA knock-down (KD) efficiency (Supplementary Figure 11C). The dependency was particularly severe for siZNF180 samples, where siZNF180 and siZNF347 had mild to negligible dependency. In order to systematically mitigate the dependency on KD efficiency, we discared samples with relatively low KD efficiency, and utilized the remaining samples to identify final DEG signatures by contrasting to NTC (highlighted in red in Supplementary Figure 11B: 2, 2 and 1 samples remained for siPPP1R2, siZNF347 and siZNF180 respectively). Given the low number of samples impeding the statistical power for DEG analysis, we applied a liberal significance threshold of nominal p-value < 0.1 with fold change > 1.2 to extract the DEG signatures per target gene.

We systematically tested if differential expressions from siRNA-DEG signatures tend to closely interact with the respective target genes by a non-parametric and threshold-free approach, Rank-Rank Hypergeometric Overlap (RRHO) test[12]. Briefly, RRHO evaluates statistical significance of overlaps between two ranked gene lists by, for instance, fold changes in differential expression from an experiment, and successively measuring the statistical significance of the number of overlapping genes[12]. Using RRHO, we tested enrichments of closely interacting genes with target genes in pSKCM network with respective siRNA-DEGs. Proximity between the target gene and another gene in the network was defined by the shortest path distance[13, 14] where a link (i.e. $l_{ij}$) distance, $d(l_{ij})$, is defined by 1-$|\rho_{ij}|$. The differential expressions were ranked by −log10(p-value) x sign(logFC), leading to positive/negative ranking score for up-/down-regulated genes in respective siRNA-DEG signatures. As shown in Supplementary Figure 3E, down-regulated genes in siZNF347 and siZNF180 are significantly enriched in proximity of *ZNF347* and *ZNF180* in pSKCM. On the contrary, up-regulated genes in siPPP1R2 are enriched in proximity of *PPP1R2* in pSKCM network.

### *EVALUATION OF TARGET NOMINATION STRATEGY BY NETWORK CONNECTIVITY*

Gene networks provide important functional contexts (e.g., modules) in which member genes operate. A network regulator can potentially regulate many other genes in the network. While the traditional differential expression and prognosis analyses offer differentially expressed and prognostic gene signatures, neither of them is capable of capturing gene-gene interactions or high-order organizational structures (e.g., coexpressed gene modules), let alone key regulators.

To understand the relationship between the differential expression and prognostic analyses and the network approach (e.g., MEGENA), we set out to test how gene network connectivity is correlated with gene differential expression and gene prognostic significance. From the pSKCM cohort, we calculated

network connectivity (i.e., the number of direct links) for each gene from the MEGENA based coexpression network and prognostic significance of each gene through survival analysis. From the single cell transcriptome (GSE72056), we determined gene differential expression between each cell cluster and the rest clusters. We then used the following regression model to evaluate their relationship:

$$\log(k) \sim \alpha * HR + \sum_{i=1}^{N} \beta_i * \log(FC_i)\,, \qquad (1)$$

where, k is the network connectivity for a gene $x$, HR is the hazard ratio from the univariate Cox proportional hazard model for the gene *x*, and $FC_i$ (Fold Change) is the raito of the average expression level of the gene *x* in a cell cluster *i* to that of *x* in the rest clusters.

From this model, we calculated the proportions of the variance in the network connectivity explained by prognostic significance and differential expression. Prognostic significance accounts for a very small proportion (0.022%) of the variance in the gene network connectivity. While differential expression in only 4 out of the 15 cell clusters can explain more than 20% variance in the network connectivity, differential expression in the M1-macrophage cluster (CLS3) can only explain 2.84% of the variance in the gene network connectivity. Therefore, network connectivity is distinct from the differential expression and prognostic significance.

Furthermore, we sought to evaluate if the combination of survival analysis of bulk tissue RNA-seq data and differential expression analysis of single cell transcriptome can identify key targets such as *MYO1F* and *ZNF180*. Marker genes of a cell cluster were identified by differential expression analysis with FDR $< 0.05$ and fold change $> 2$. We then intersected each cell cluster marker gene signature with the prognostic gene signature from the pSKCM cohort. The good prognosis gene signature (GOSG) share 150 genes with the markers of the CD8+ T-cell cluster (CLS11) (FET p=1.09E-57) and 84 genes (including *MYO1F*) with the markers of the M1-macrophage cluster (CLS3) (FET p=4.49E-38). This is expected as the significant ovelap reflects the extent of immune infiltration in melanoma tumor, a known predictor for patient survival. But such candidate lists are still long and it is difficult to further prioritize these candidates in an unbiased manner without knowledge about their disease-specific regulatory relationship. On the other hand, this combined analysis failed to identify key protumorigenic genes such as *ZNF180*, *ZNF347* and *PPP1R2* identified by MEGENA. These findings further demonstrate that the gene network analysis complements survival analysis and differential expression analysis, thus supporting the utility of network connectivity.

We have also systematically evaluated our target prioritization strategy by intersecting the poor prognosis gene signature (POSG) and the network hubs. The resulting regulators are more likely to be essential for melanoma cell viability, based on the CRISPRi screening data in the Achilles database[15], than the poor prognosis signature alone (Wilcox p-value=3.03E-4; Supplementary Figure 10).

In conclusion, these results strongly demonstrate that network connectivity provides critical information that complements differential expression and prognostic significance, and integration of the network analysis and the survival analysis leads to the identification of biologically more important regulators. More importantly, the network analysis provides functionally relevant network contexts (e.g., modules) in which member genes operate, and such functional contexts cannot be developed through the traditional differential expression and survival analysis.

### *DIFFERENTIALLY EXPRESSED PATHWAYS BY ZNF180 SUPPRESSION IN SKMEL147 CELLS IN VITRO*

We performed enrichment analyses of siZNF180-DEG signatures (nominal p-vale < 0.1, fold change > 1.2) in pSKCM modules. Distinct branches of MEGENA module hierarchy were simultaneously hit by siZNF180-DEG and pSKCM survival (i.e. POSG/GOSG) signatures (FET FDR < 0.05). Within these modules, we elucidated functions and pathways by intersecting with MSigDB database[16], and intersected with protein interactions curated by STRING database with high confidence interactions (confidence score > 0.7) [17]. These results are summarized in **Supplementary Table 1**.

# SUPPLEMENTARY TABLES

**Supplementary Table 1.** Summary of functions and key genes associated to pSKCM modules significantly enriched by siZNF180-DEGs in **Figure 4H**. Module names are color-coded in accordance to enrichments of MSH2, PAI-1 correlation signatures, and siZNF180-DEG signatures depicted in **Figure 4H**. The following font colors capture respective enriched signatures: **Blue** fonts = siZNF180-DN and POSG, **Red** fonts = siZNF180-UP and GOSG, **Cyan** fonts = siZNF180-DN and PAI-1(-), **Black** fonts = siZNF180-DN, PAI-1(-) and MSH2(+) signatures.

| **Module** | **Top 10 hubs** | **Associated Pathways** | |
|---|---|---|---|
| *Poor Prognosis Genes, down-regulated by siZNF180* | | *MSigDB (FET FDR/EFC)* | *Key genes in STRING protein network* |
| **M198** | *EXOC5, NAA30, G2E3, FANCM, TMX1 (ZNF180)* | ATCATGA,MIR-433 (1.74E-3,16.5) | DNA repair and ubiquitination (FANCM[18]) |
| **M205** | *KRIT1, TMEM106B, LUZP6, PNPLA8, CCDC132, ZNF680, ZNF12, MKLN1* | NA | E3 ligase (HERC5[19],UBE3C[20],CUL1[21]) |
| **M257** | *C4orf21, PLK4, KIF15, KIAA1524, DEPDC1, CCNA2, CCNE2, ATAD5, GTPBP10* | HALLMARK_G2M_CHECKPOINT (9.58E-14,13.0) | CDK1 driven cell cycle (CDK1,CCNA2, KIF15)[22] |
| **M25** | *PRPF40A, SMEK2, ZNF148, XPO1, ZBTB11, DHX36, BZW1, SF3B1, SFRS3, CUL3* | HALLMARK_MYC_TARGETS_V1 (9.43E-4,2.58) | oncogenic kinases/Rho kinase, GTPases (PIK3CA[23], ROCK1, RHOA, RAB1A, RHOQ)[24], splicing (SFRS13A, SRSF10, DDX5)[25-27], actin filaments/microtubules (ACTRs) |

| | | | |
|---|---|---|---|
| **M30** | *LRRC40, TRUB1, ZNF644, CDC73, PKN2, FAM175B, ARCN1, RNF214, YME1L1, WAPAL* | UBIQUITIN_CYCLE (5.56E-4,4.18) | histone variant in double-strand break repair (H2AFX)[28], chromatin modification (WAPAL)[29], ubiquitination (UBE2D1[30], RPF1[31]) |
| **M680** | *KIAA1712, SMARCAD1, CUL5, SNRNP48, ZNF322A, ZRANB2, PRPF4B, KIAA1731* | DOUBLE_STRAND_BREAK_REPAIR (2.37E-2, 22.1) | RNA splicing (PRPF4B-ZRANB2)[32, 33], DNA repair (ZNF180[34], ATM/ATR[35]) |
| **M11** | *RNF160, CTDSPL2, DNAJB14, PPP1R12A, LIN54, SMARCA5, IREB2, SFRS2IP, DHX15, HAUS3* | BIOPOLYMER_METABOLIC_PROCESS (3.70E-4, 1.59) | chaperone (DNAJB14[36], HSPAs[37]) / proteasome(PSMA3/4, PSMC6) RNA, DNA polymerases (POLA1[38], POLR2B[39])/pre-mRNA splicing (DHX15[40]) / ubiquitination (RNF160[41], UBR1[42]) / chromatin remodeling (SMARCA5[43], ARID4A[44]) |
| **M28** | *BDP1, POLK, TTC37, EFCAB7, ZNF182, AFF4, VPS4A, DCTN4, SRFBP1, C5orf24* | BIOPOLYMER_METABOLIC_PROCESS (2.45E-3, 1.76) | ubiquitination (UBE2s) |
| **M24** | *REST, SBNO1, RIF1, GMCL1, PPP4R2, UHMK1, CCNT1, ZNF192, HNRNPR, IL6ST* | BIOPOLYMER_METABOLIC_PROCESS (1.78E-7, 1.66) | oncogenesis (KRAS[45], ROCK2[24], CTNNB1[46], TP53[47]) |
| *Good Prognosis Genes, up-regulated by siZNF180* | | | |
| **M170** | *BRMS1, C11orf59, PPP1CA* | NA | tumor suppressor (BRMS1[48]) |
| **M39** | *ZC3H3, SHARPIN, HSF1, MAF1, GLI4* | NA | |
| **M42** | *GADD45GIP1, TRAPPC5, CLPP, NDUFB7, CCDC94, ELOF1, NDUFA11,* | INTRACELLULAR_TRANSPORT (6.69E-3, 4.22) | negative regulator of cell cycle (GADD45GIP1[49]), mitochondrial membrane respiratory chain (NDUFAs/NDUFBs) |

| | | | |
|---|---|---|---|
| | *SIRT6, C19orf70* | | |
| **M46** | *MRPL41, PTRH1, C9orf142, SSNA1, ARRDC1, C9orf16, NACC2* | CYTOPLASMIC_PART (8.99E-4, 3.09) | |
| **M48** | *DOHH, ZNF205, ZNF414, ZNF628, ZNF653, REXO1, CCDC124, MAP2K2, PLSCR3, C19orf22* | NUCLEUS (2.31E-3, 1.93) | epigenetic transcription control (MBD3[50], SMARCA4[51])/ubiquitin (UBA52), protein phosphorylation with anti-tumor activity (PRKACA[52])/RNA splicing (RNPS1[53]), post-translational modification (DOHH[54]) |
| **M62** | *C11orf68, SART1, RELA, BANF1, WDR74* | HALLMARK_DNA_REPAIR (3.77E-2, 7.41) | RELA driven pathway |
| **M339** | *TSSC4, MOB2, PHRF1* | SULFURIC_ESTER_HYDROLASE_ACTIVITY (2.85E-2, 65.8) | |

# SUPPLEMENTARY FIGURES

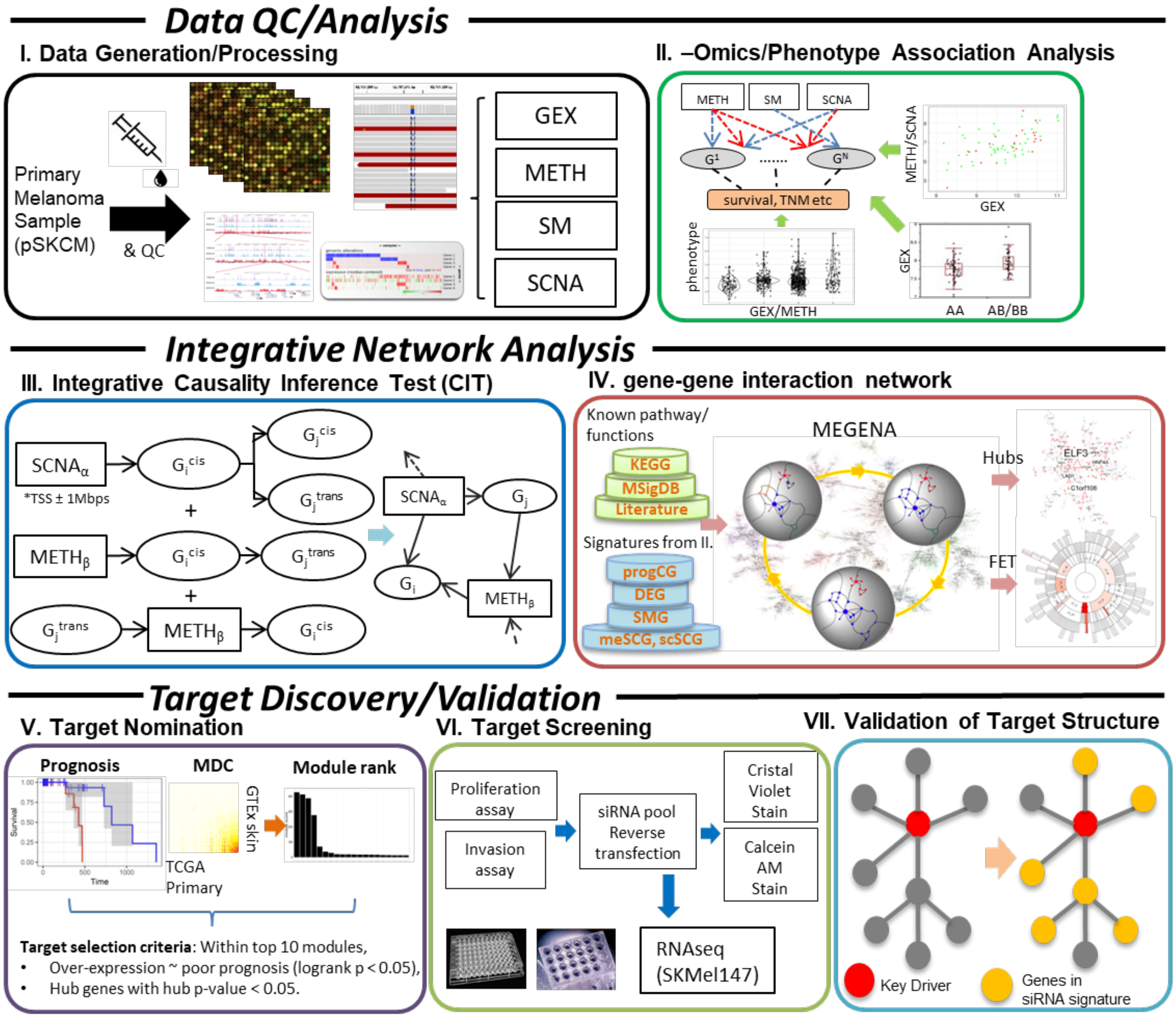


**Supplementary Figure 1. Illustration of overall study design applied to –omics data from TCGA primary tumors of subcutaneous melanoma (TCGA-pSKCM)**. The study is mainly composed of three components of Data QC and analysis, Integrative Network Analysis, and Target Discovery and Validation. Data QC/Analysis: I) Illustration data types and Quality Controls (QC) applied to gene expression (GEX), methylation (METH), somatic mutations (SM), and somatic copy number alterations (SCNA). II) Illustration of association analysis gene expression and multiple –omics data (METH, SCNA and SM) with clinical phenotypes such as overall survival outcome and TNM staging. Integrative Network Analysis: III). Construction of integrative causal networks by utilizing causality inference test (CIT) framework to integrate alterations in METH and SCNA with GEX. IV) Construction of gene-gene interaction network by Multiscale Gene co-Expression Network Analysis (MEGENA). Various gene signatures curated from public domain and within TCGA-pSKCM cohort are projected to the network to identify significantly enriched/associated modules. Target Discovery and Validation: V). Target nomination: modules are first filtered by comparing interactions to normal skin from GTEx, and then ranked according to their associations to overall survival. Hub genes in top ranked modules with

prognostic significance are nominated. VI) <u>High-throughput siRNA screening of nominated targets</u> on SKmel147 and A375 cells for invasion and proliferation screening. Samples showing promising screening results are subject to RNA-sequencing for gene expression profiling. VII). <u>Differentially Expressed Genes (DEGs) from RNA-sequencing data from siRNA samples</u> are projected to network neighborhood of the target genes to validate gene-gene interactions inferred by MEGENA.

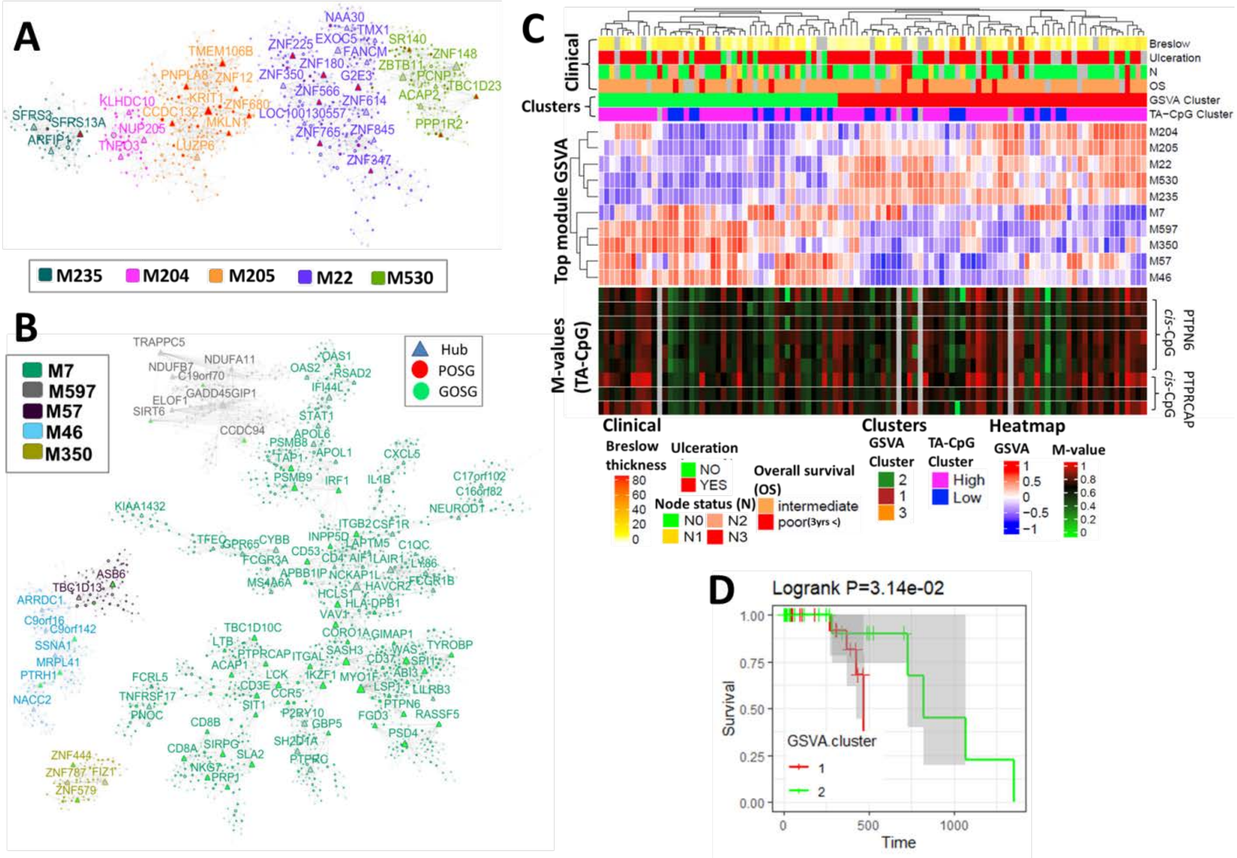


**Supplementary Figure 2. A**. Top subnetworks enriched for good prognosis associated genes in pSKCM (POSG). Different modules are color-coded in labels and borders as shown in the top left legend. Hub genes and prognosis associated genes are highlighted by node shapes and node fill color as shown in bottom left legend. **B**. Top subnetworks enriched for poor prognosis associated genes in pSKCM (GOSG). Hubs and modules are highlighted accordingly. **C**. Heatmap of sample-wise module enrichments by GSVA score (middle). Top colorbars depict key clinical features including Brewslow thickness (Breslow), Ulceration, node status (N), death observed within 3 years follow up (OS), and samples clustered by GSVA score (GSVA cluster) or methylation in cis-CpG sites of *PTPN6* and *PTPRCAP*. The bottom heatmap shows methylation profiles of the cis-CpG sites. **D**. Kaplan-Meier plot of pSKCM patients' overall survival, segregated by GSVA clusters in **C**. The logrank p-value at the top was obtained from two-sided logrank test.

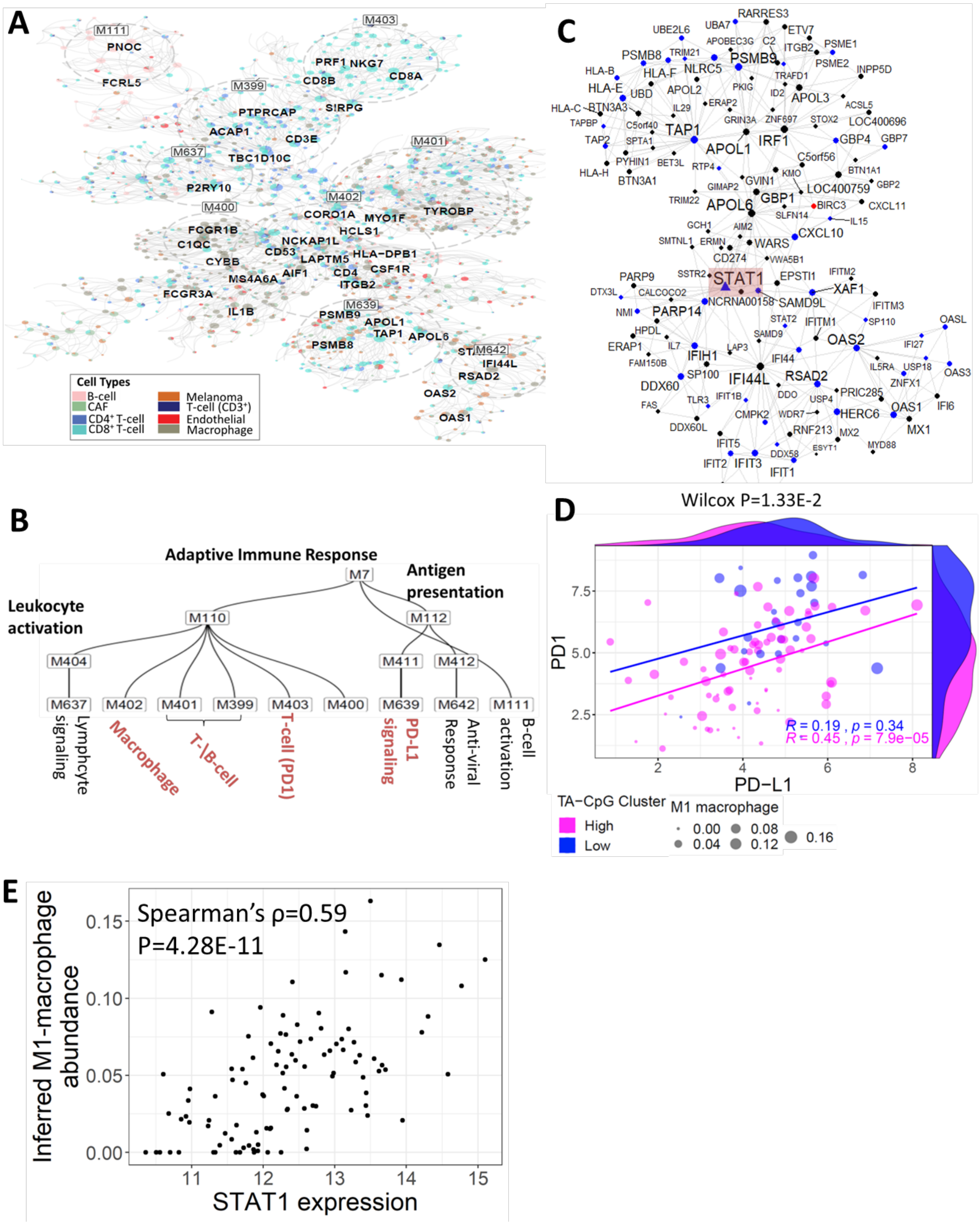


**Supplementary Figure 3. Dissection of immune response signaling in primary melanoma micro-environments**. **A**. Subnetwork of immune response module, M7. Dashed borders mark child modules, and module hierarchy is shown in **B**. Hub genes are labeled with respective gene symbols. The piechart nodes summarize cell types expressing the respective genes in GSE72056 scRNA-seq data. **B**. Parent-child module hierarchy within M7. Representative pathways are labeled. **C**. Down-regulated genes (blue)

in *Stat1* knock-out murine bone marrow-derived macrophages are enriched in the 3-layer neighborhood of STAT1 in pSKCM network. **D**. Differential correlation between PD1 and PD-L1 expressions within TA-CpG High or TA-CpG Low cluster. Differential expressions of individual genes are depicted in density plots on the borders, and the significant differences are shown by two-sided Wilcoxon test p-value. Point size describes CIBERSORT inferred M1-macrophages in pSKCM. **E**. Correlation between log-normalized STAT1 expression and inferred M1-macrophage abundance in pSKCM cohort. The Spearman correlation coefficients and p-value from two-tailed test are shown in the plot.

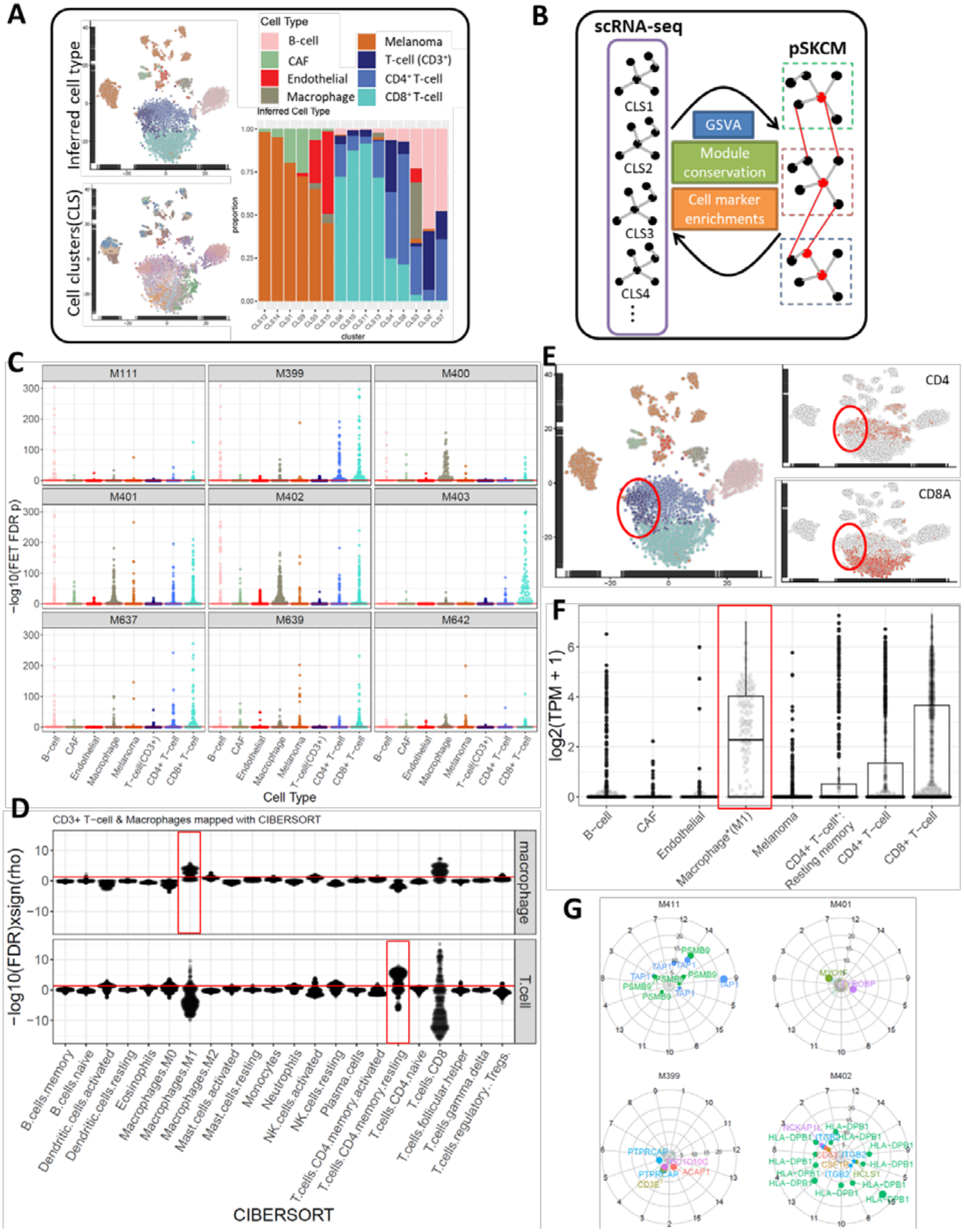

**Supplementary Figure 4. Overview of GSE72056 scRNA-seq analysis. A.** Cell population inference: By supervised approach with established cell type markers, cell types of individual cells are inferred (top

left). Cells are further classified by unsupervised clustering approach (bottom left), annotating each cell cluster with varying compositions of different cell types (right). **B.** Cell-type specific interactions: gene interactions from TCGA-pSKCM (right) are compared to cell-cluster specific interactions (left). **C.** Cell types enriched for individual gene expression in scRNA-seq. Each dot is a gene belonging to the specified modules in each box. One-tailed FET with odds ratio > 1 were performed to test enrichment of cells expressing a gene in cells from each cell type. The x-axis is inferred cell types in scRNA-seq data. the y-axis is –log10(FDR corrected FET p-value) for the enrichment of cells expressing each gene in the cell types. **D**. Correlation analysis between CIBERSORT inferred cell abundance (x-axis) and CPM inferred cell abundance. Each dot represents a cell from the scRNA-seq data, whose relative abundance in the pSKCM was inferred by the CPM algorithm. Y-axis, –log10(Spearman correlation FDR) x sign(correlation coefficient), denotes the correlation between individual cell abundance score by CPM and cell type score by CIBERSORT. Upper panel: Macrophage abundance correlated to M1-polarized macrophage abundances. Lower panel: CD3+ CD4+ CD8- T-cells correlated to resting memory CD4+ T-cells. **E**. CD4/CD8 marker expressions (CD4/CD8A) on T-cell subpopulation highlighted in red circle. Left panel: highlighted CD3+ T-cell subpopulation with unknown T-cell subtype. Top right panel: These cells exhibit moderate CD4 expressions. Bottom right panel: These cells do not show CD8 expression. **F**. M1 macrophage-specific expression of *MYO1F* in scRNA-seq data (185 M1-macrophage cells from the total of 4,645 cells). The borders of boxplots show the lower quantile, median and upper quantiles, and whiskers span towards the minima and maxima. **G**. The radar plots show interaction partners of several hub genes in M7 significantly overlap with those from ct-networks (radial axis: -log10(FET FDR) of pSKCM interaction partners in ct-network interaction partners, circumference axis: cell clusters from published melanoma single-cell transcriptome[16].

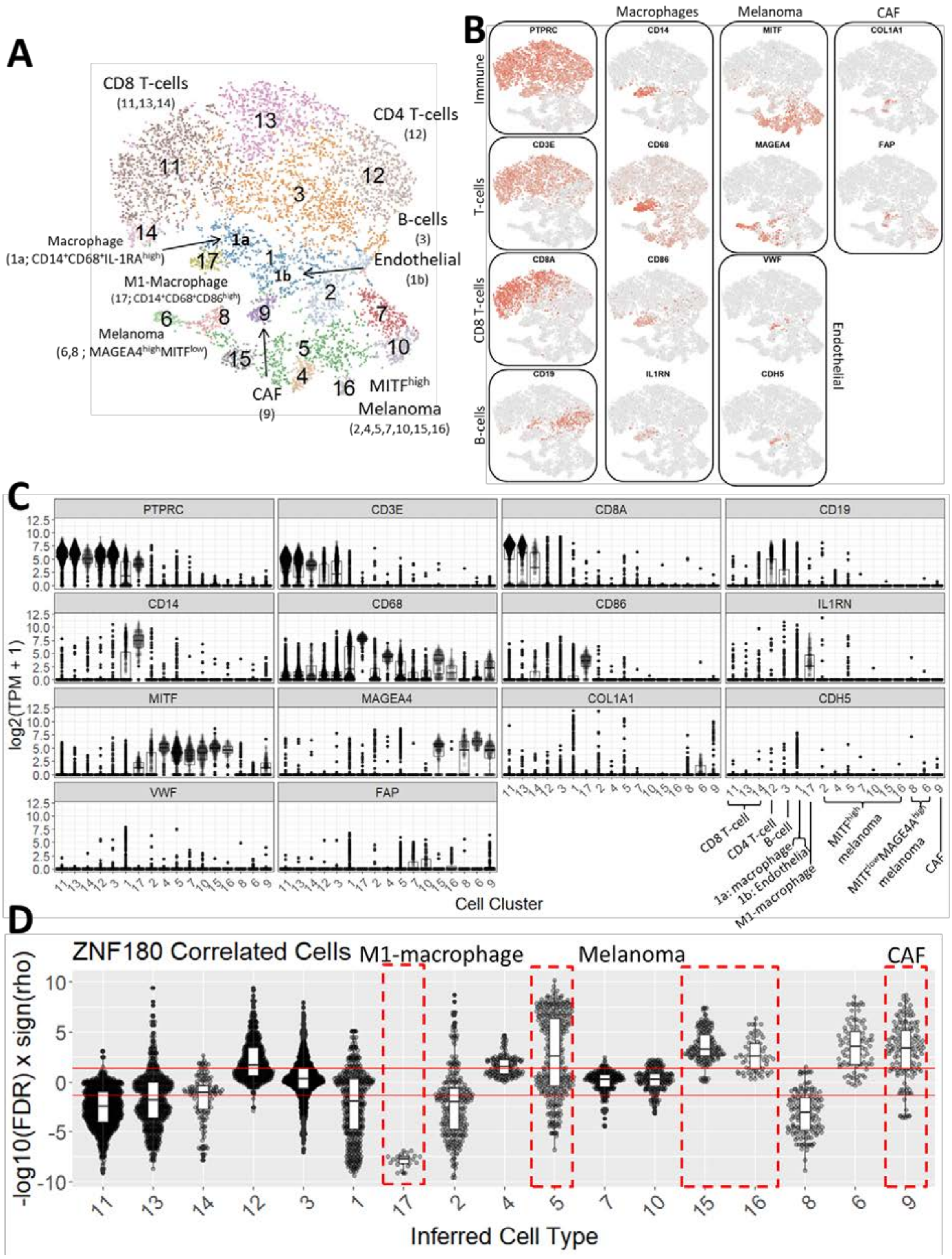


**Supplementary Figure 5. Cell type analysis of Jerby-Arnon et al 2018 (scRNA-seq) data. A.** tSNE plot of scRNA-seq. Cell clusters are labeled in different colors, along with respective cell types. Each cell

type label is tagged with respective clusters in the bracket. **B**. tSNE plot showing marker gene expressions of cell types in  A. **C**. Expression of marker genes across 7,16 cells in different clusters. Each dot is one cell expressing respective genes. Per gene, the boxplots show the lower quantile, median and upper quantile expressions within each cell cluster, and the whiskers span to the respective minima and maxima. **D**. Cell abundances correlated with *ZNF180* expressions in pSKCM. Cell abundances were inferred for 7,168 cells across 103 pSKCM bulk samples by scBio package, and each inferred abundances were tested for correlations with ZNF180 expressions in pSKCM by Spearman's correlation. Each dot is one cell from the Jerby-Arnon *et al* 2018 scRNA-seq. The correlation p-values were adjusted by FDR, and y-axis reflects −log10(FDR) signed by correlation direction from Spearman's correlation. Melanoma cells (clusters 5,15,6, and 7) tend to increase with higher *ZNF180* expressions. Dotted red rectangles highlight cell clusters tracking with *ZNF180.* The borders of boxplots show the lower quantile, median and upper quantile within each cell cluster, and the whiskers span to the respective minima and maxima.

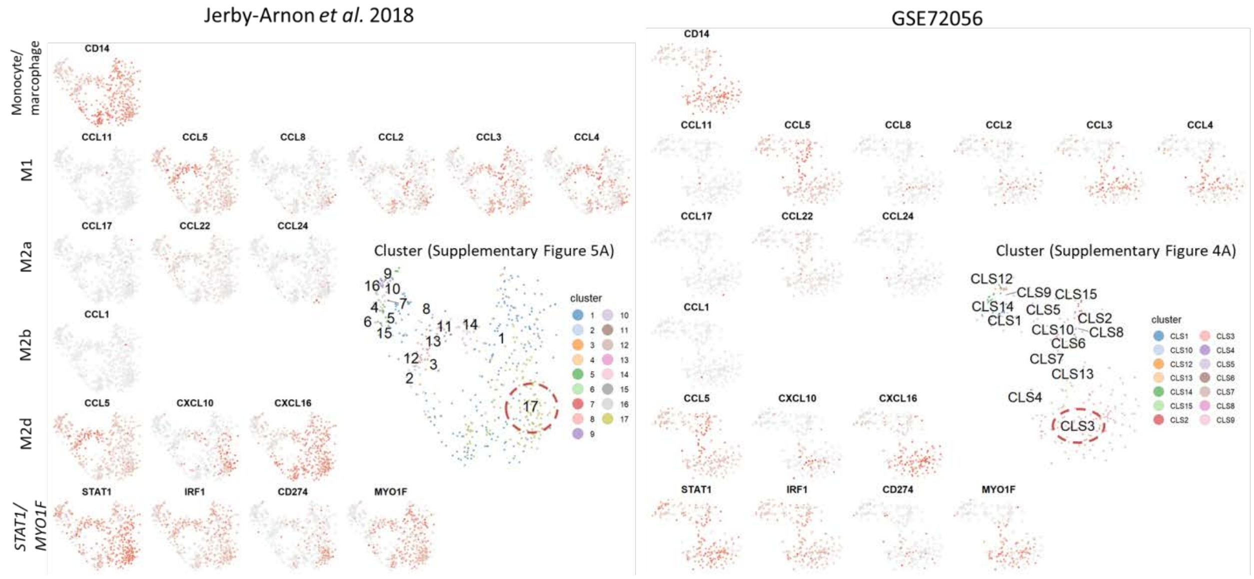


**Supplementary Figure 6. Chemokines as markers of macrophage polarization.** CD19+ cells were subset and various macrophage markers were plotted for GSE71056 (right) and Jerby-Arnon *et al*. 2018 (left). tSNE plots showing the original cell cluster assignments from the full data are embedded. Cell cluster corresponding to M1-macrophages is highlighted by red circle. The first row shows expression of CD14, a marker of monocyte/macrophages. From second to fifth row, each row depicts unique chemokines secreted by macrophages with different polarizations. The markers were obtained from Rozer 2015[55]. The sixth row shows key genes from *STAT1/MYO1F* axis proposed in this study.

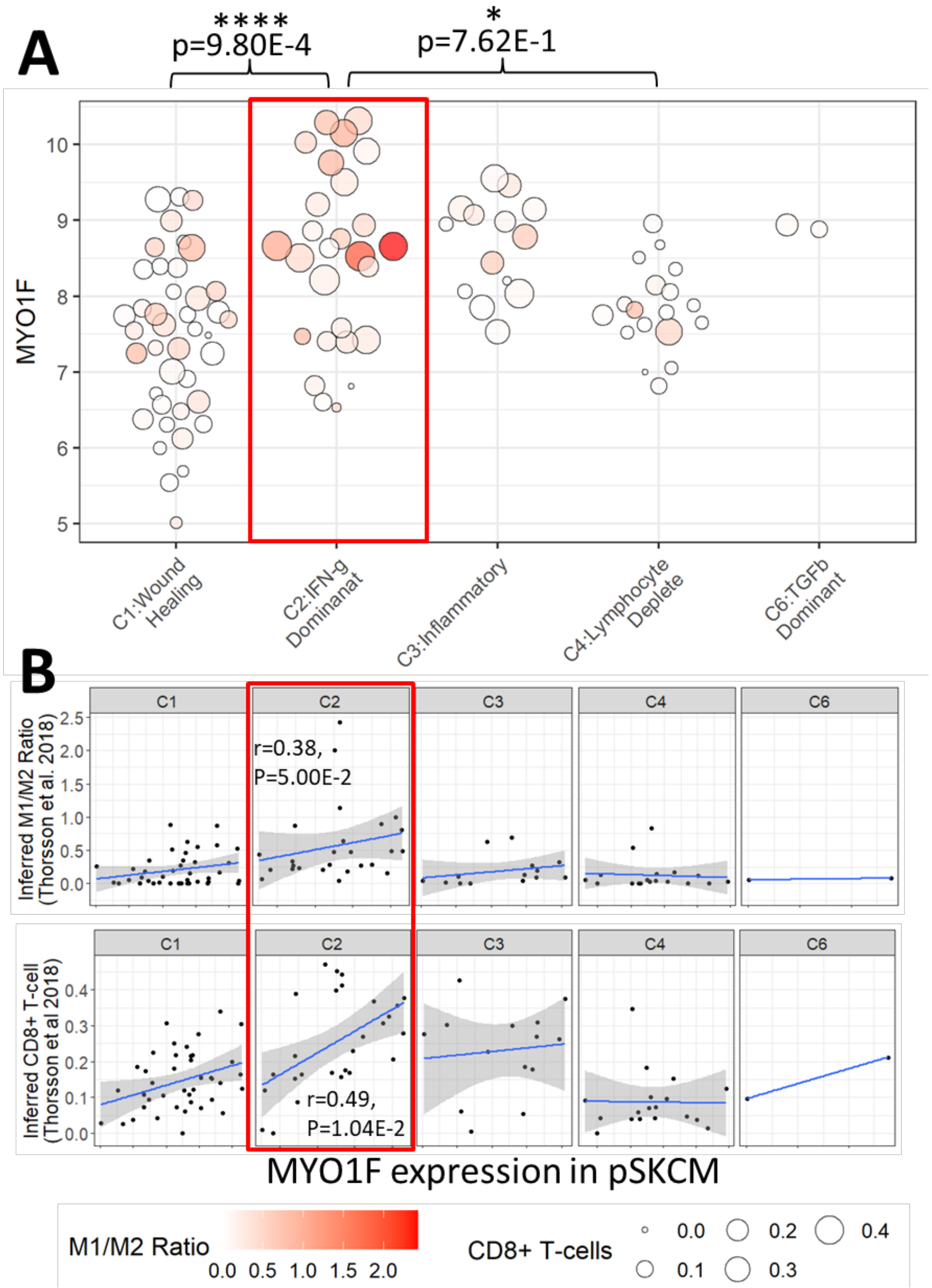


**Supplementary Figure 7. MYO1F expression in pSKCM immune subtypes in Thorsson et al. 2018. A.** Scatter plot between the immune subtypes and *MYO1F* expressions in pSKCM. The point colors correspond to M1/M2 macrophage ratio, and sizes correspond to CD8+ T-cell abundances as inferred in Thorsson et al. 2018, and are illustrated in the bottom legend. Significant differences via two-wided Wicoxon test p-values are highlighted at the top. **B**. Immune subtype specific correlations between MYO1F and M1/M2 ratio, or CD8+ T-cell abundances. Subtype C2 is the only subtype showing the significant correlations. The 95% confidence intervals for Spearman's correlations with MYO1F expressions are - Inferred M1/M2 ratio: (2.5E-3, 0.67), Inferred CD8+ T-cells: (0.13, 0.73). The estimates and respective p-values are shown in the figure.

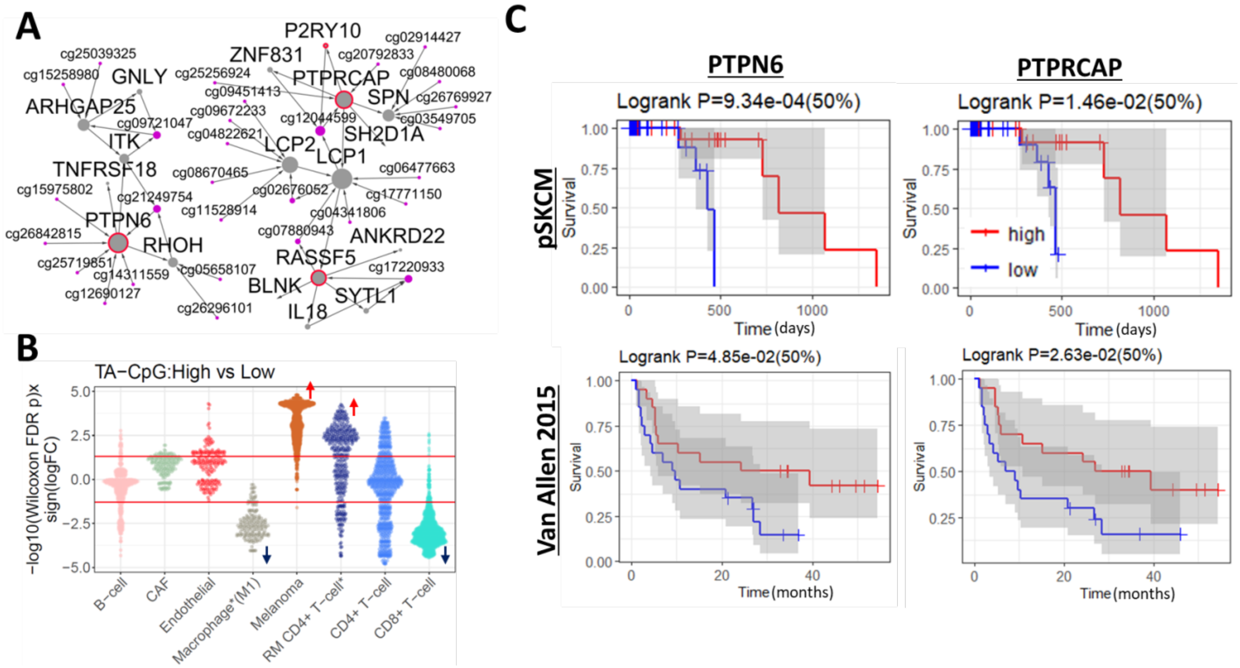


**Supplementary Figure 8. A.** Integrative causal network of methylation and gene expression in pSKCM. Key network drivers in this network that overlap with pSKCM network hubs are highlighted by magenta. **B**. Differential cell abundances between TA-CpG High and TA-CpG Low clusters. Cell abundances were inferred by scBio package, and each dot is one cell from the GSE72056 scRNA-seq data. **C**. Kaplan-Meier plots for median-high and median-low expressions of *PTPN6* and *PTPRACAP* in pSKCM and Van Allen *et al* 2015 cohorts. Logrank p-values from two-sided logrank tests are also specified on top.

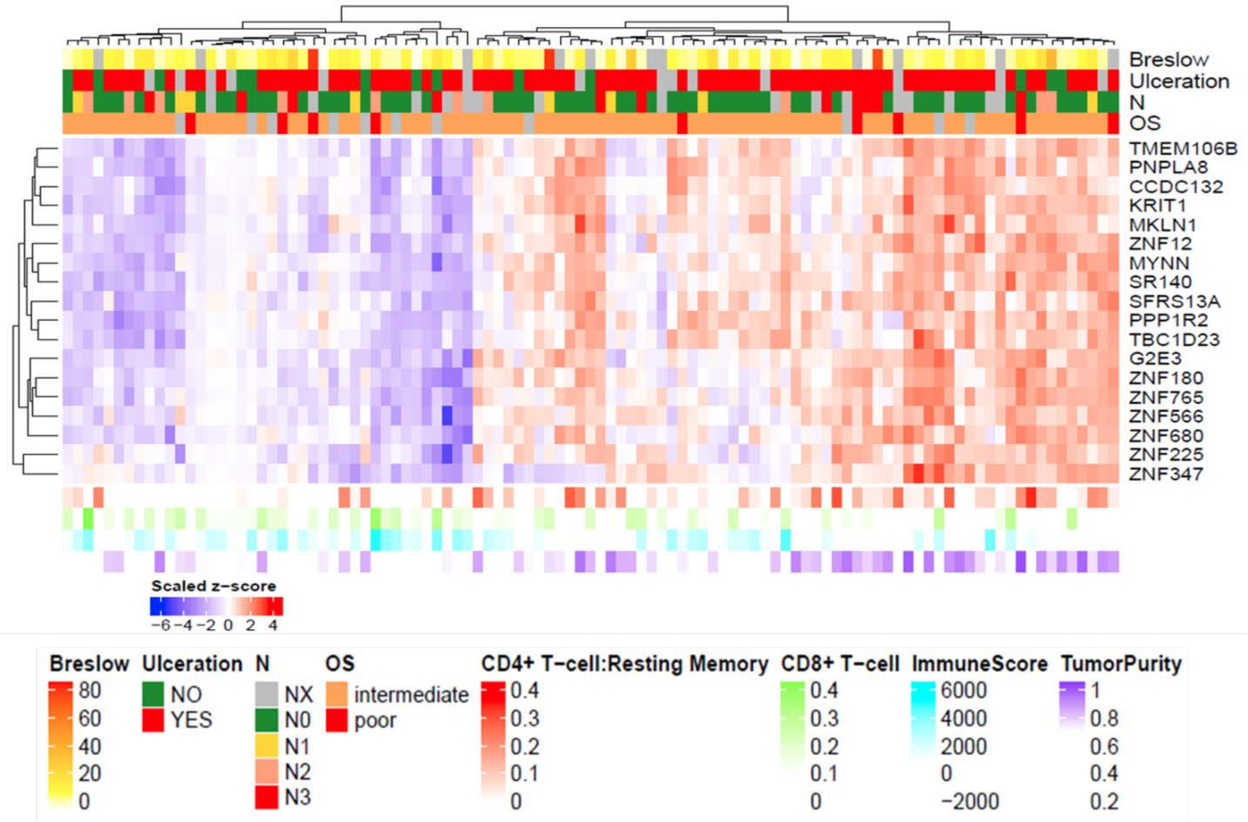


**Supplementary Figure 9.** Center: Heatmap of 18 nominated targets in **Figure 3A**. Top: key clinical traits including Breslow thickness, ulceration, node status and overall survival discretized by poor outcome (death within 2 years of follow-up) or intermediate outcome (death between 2 and 5 years of follow up). Bottom: inferred cell type abundance scores from ESTIMATE (ImmuneScore, TumorPurity) and CIBERSORT (CD8+ T-cell, resting memory CD4+ T-cell). Only the correlated cell types to the 18 gene expressions are shown.

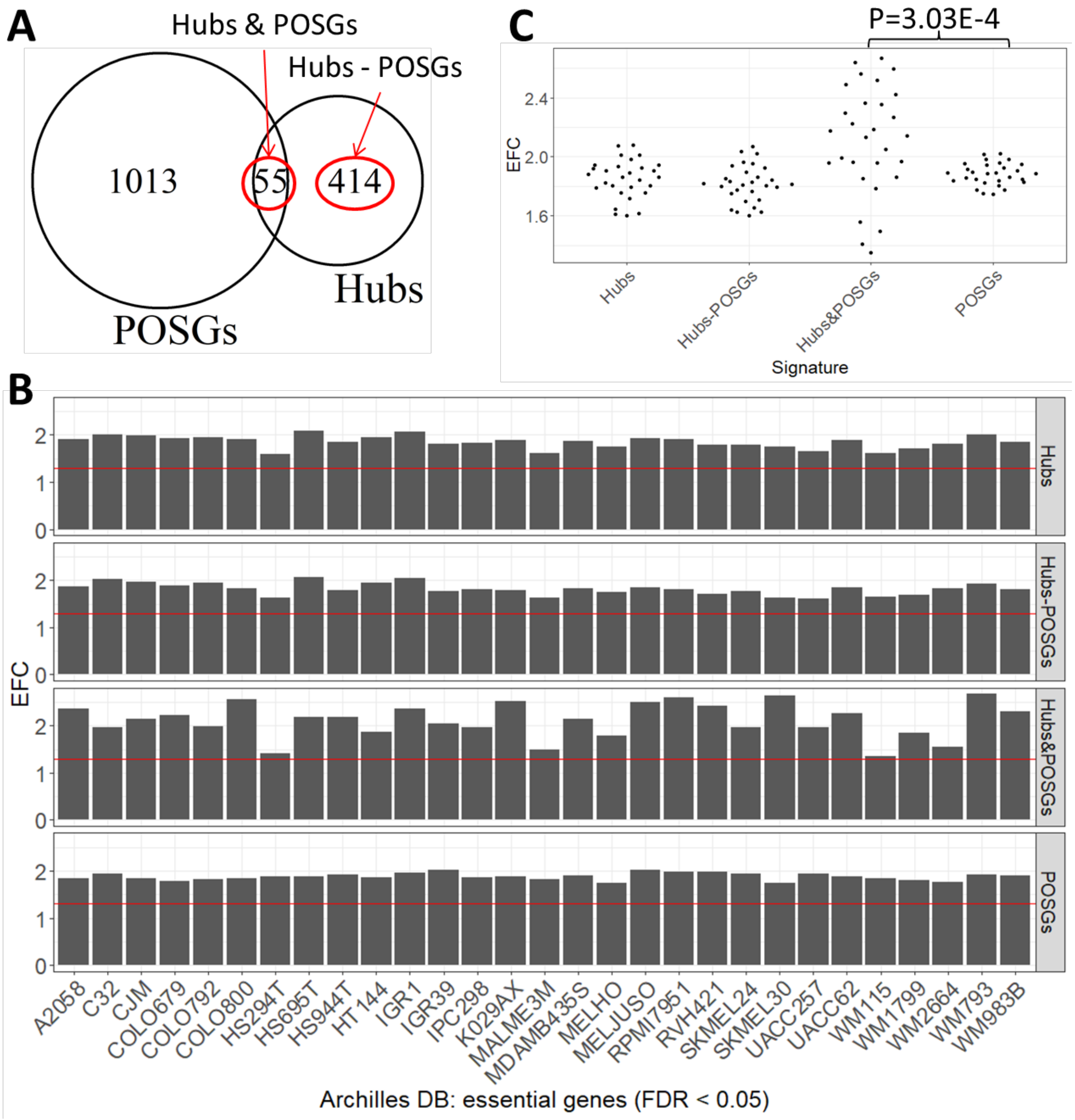


**Supplementary Figure 10. Validation of candidate target nomination approach by POSGs and hubs. A.** Venn diagram of all POSGs in pSKCM and network hub genes. **B**. Enrichment fold-change (EFC) of the essential genes of melanoma cells, identified by CRISPRi screening (FDR < 0.05) from Achilles database[15]. Each panel represents different signature from the Venn diagram in (**A**). **C**. Scatter plot of enrichment fold-changes of different signatures for the essential genes in different melanoma cells. The two-sided Wilcox test p-value showing significant difference in EFC between 'Hubs & POSGs' and 'POSGs' signatures are marked at the top.

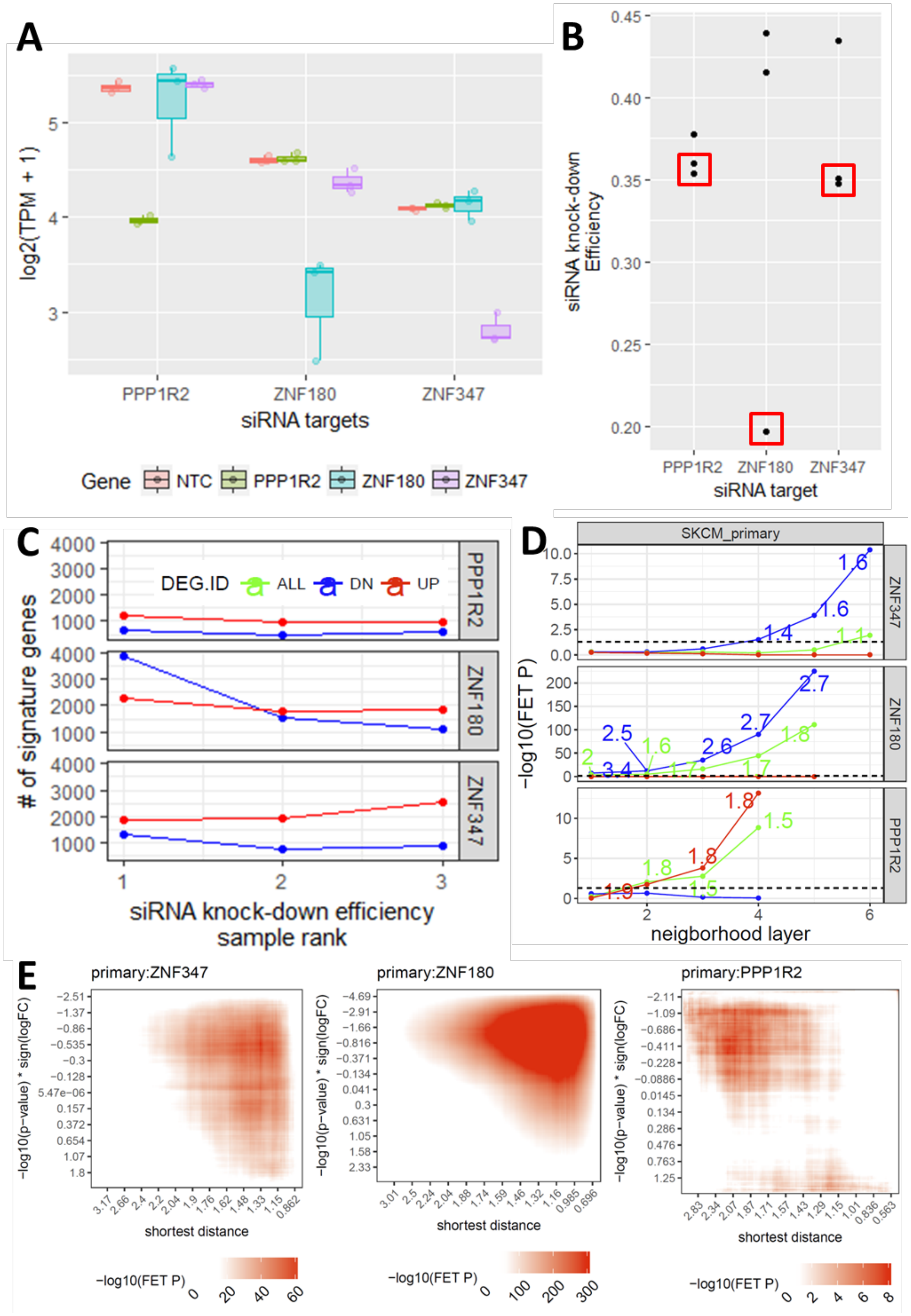


**Supplementary Figure 11.** Validation of TCGA-pSKCM co-expression network topology surrounding top nominated targets, *PPP1R2*, *ZNF347* and *ZNF180*. **A**. Gene expressions of the targeted genes in

siRNA-transfected cells (3 replicates per siRNA). X-axis specifies the respective target genes suppressed in the panel. Different colors highlight samples transfected by siRNA and non-transfected controls (denoted NTC). The boxplots show the lower quantile, median and upper quantile within each siRNA, and the whiskers span to the respective minima and maxima. **B.** Knock-down efficiency of siRNA transfections for target genes specified on the x-axis. **C**. Dependency of the size of differentially expressed gene (DEG) signatures with nominal p-value < 0.01, on the selection of siRNA-transfected samples by knock-down efficiency in **B**. **D**. Enrichment of DEG signatures per targeted gene in the *l*-layer network neighborhood of the respective gene in TCGA-pSKCM co-expression network by one-sided Fisher's Exact Test with odds ratio > 1. X-axis is the number of l-layer to define the network neighborhood, and y-axis is –log10(FET p-value) for significance of the DEG signature enrichment. Up-/down-regulated DEG signatures (denoted UP/DN) are highlighted in red/blue, and combination of up- and down-regulated genes (denoted ALL) is highlighted in green. **E**. Heatmap of –log(FET p-value) for enrichment of the DEG signatures as evaluated by one-sided Rank-Rank Hypergeometric Overlap (RRHO) test[12].

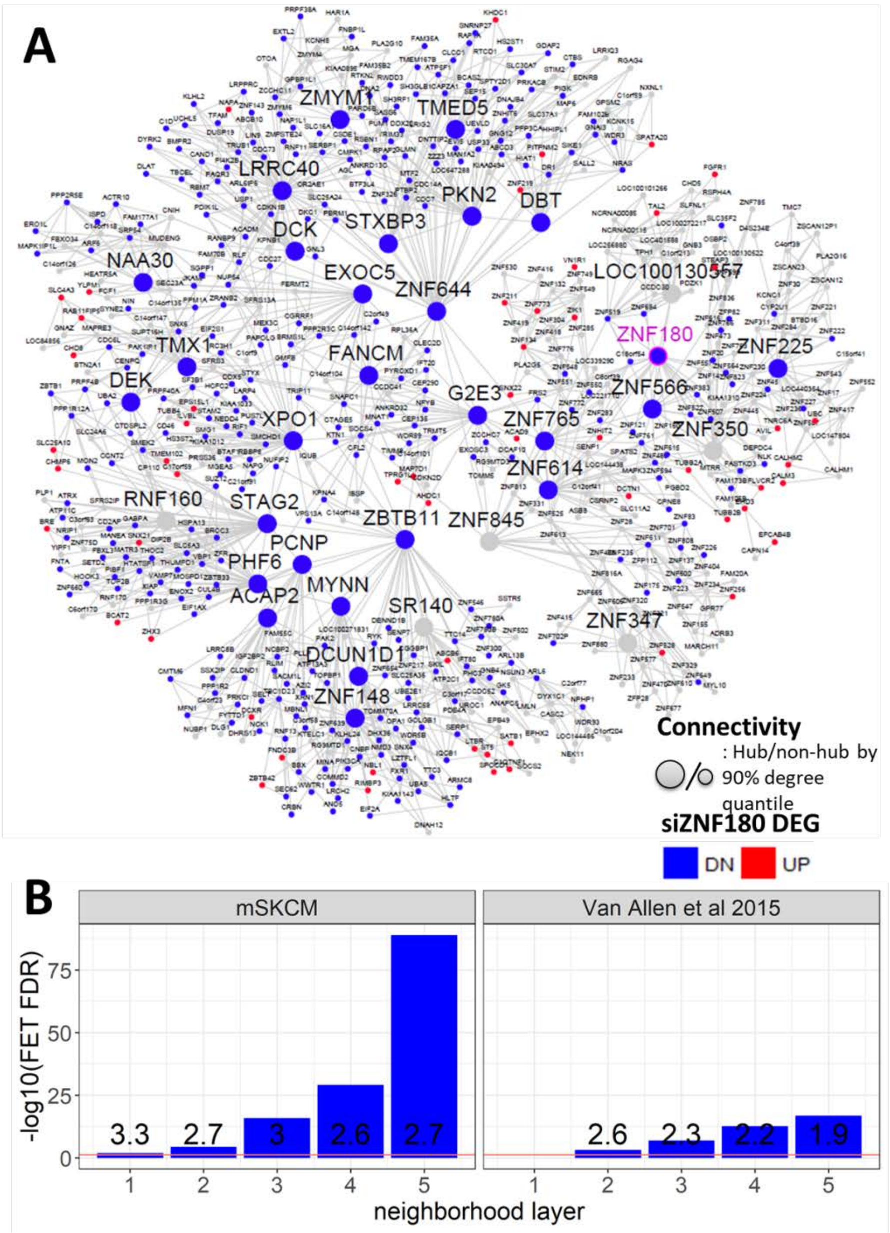


**Supplementary Figure 12. Validation of ZNF180 centered network by differentially expressed genes upon siZNF180 transfection. A.** 4-layer neighborhood of *ZNF180* in pSKCM network is enriched by siZNF180-DN signature. *ZNF180* is highlighted in magenta. **B**. Enrichments of siZNF180-DN signature in co-expression networks from two independent bulk cohorts, mSMCK and Van Allen *et al.* 2015.

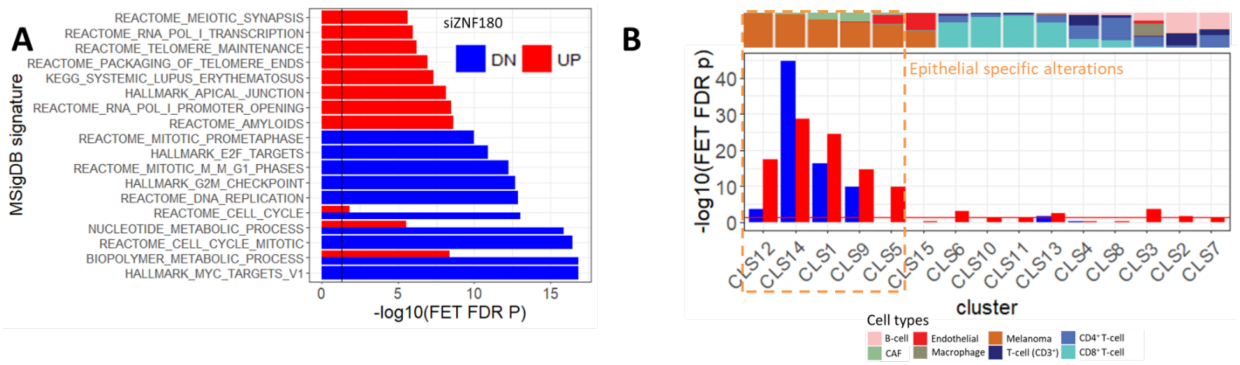


**Supplementary Figure 13.** Enrichments of siZNF180-DEG signatures by one-sided Fisher's Exact Test with odds ratio > 1, in functional (A) and single-cell (B) signatures. **A**. Summary of top enriched MSigDB functions and pathways for siZNF180-Poor-DN (blue) and siZNF180-Good-UP (red). **B**. Enrichments of siZNF180-DEG signatures in marker gene signatures of cell clusters in published melanoma single cell transcriptome.

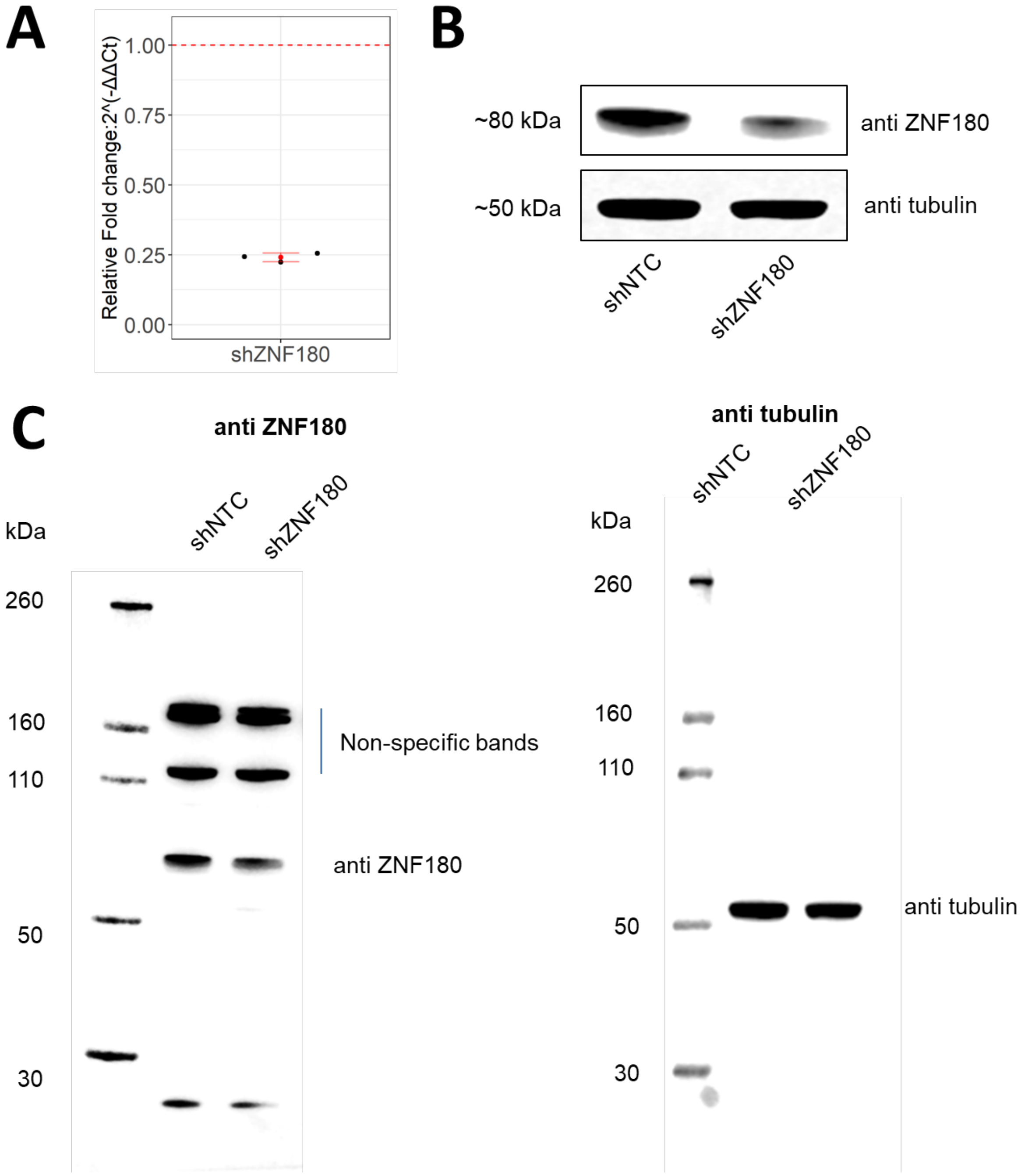


**Supplementary Figure 14.** (A) Real-time qPCR showing individual *ZNF180* expressions in shZNF180 transfected cells, relative to the control non-transfected samples (shNTC). Y-axis is fold change of *GAPDH*-normalized expressions of *ZNF180* in shZNF180, relative to shNTC by $2^{-\Delta\Delta Ct}$. The experiments were repeated three times for replication. The errorbar is presented as mean ± SD. (B) Western blot of ZNF180 levels in SkMel147 melanoma cells stably transduced with DOX-inducible PLKO-Tet-On-shNTC or PLKO-Tet-On-shZNF180. qPCR graph shows average relative expression normalized to *GAPDH*. (C) Raw images of Western blot: Antibody was purchased from Sigma and showed presence of multiple non-specific bands at higher and lower molecular weight.

## References


1. Liberzon A, Birger C, Thorvaldsdottir H, Ghandi M, Mesirov JP, Tamayo P: **The Molecular Signatures Database (MSigDB) hallmark gene set collection**. *Cell Syst* 2015, **1**(6):417-425.
2. Liberzon A, Subramanian A, Pinchback R, Thorvaldsdottir H, Tamayo P, Mesirov JP: **Molecular signatures database (MSigDB) 3.0**. *Bioinformatics* 2011, **27**(12):1739-1740.
3. Therneau TM: **A Package for Survival Analysis in S**. In., 2.38 edn; 2015.
4. Yoo S, Takikawa S, Geraghty P, Argmann C, Campbell J, Lin L, Huang T, Tu Z, Foronjy RF, Spira A *et al*: **Integrative analysis of DNA methylation and gene expression data identifies EPAS1 as a key regulator of COPD**. *PLoS Genet* 2015, **11**(1):e1004898.
5. Millstein J, Zhang B, Zhu J, Schadt EE: **Disentangling molecular relationships with a causal inference test**. *BMC Genet* 2009, **10**:23.
6. Schraven B, Schoenhaut D, Bruyns E, Koretzky G, Eckerskorn C, Wallich R, Kirchgessner H, Sakorafas P, Labkovsky B, Ratnofsky S *et al*: **LPAP, a novel 32-kDa phosphoprotein that interacts with CD45 in human lymphocytes**. *J Biol Chem* 1994, **269**(46):29102-29111.
7. Azoulay-Alfaguter I, Strazza M, Peled M, Novak HK, Muller J, Dustin ML, Mor A: **The tyrosine phosphatase SHP-1 promotes T cell adhesion by activating the adaptor protein CrkII in the immunological synapse**. *Sci Signal* 2017, **10**(491).
8. Frishberg A, Peshes-Yaloz N, Cohn O, Rosentul D, Steuerman Y, Valadarsky L, Yankovitz G, Mandelboim M, Iraqi FA, Amit I *et al*: **Cell composition analysis of bulk genomics using single-cell data**. *Nat Methods* 2019, **16**(4):327-332.
9. Dobin A, Gingeras TR: **Mapping RNA-seq Reads with STAR**. *Curr Protoc Bioinformatics* 2015, **51**:11 14 11-19.
10. Liao Y, Smyth GK, Shi W: **featureCounts: an efficient general purpose program for assigning sequence reads to genomic features**. *Bioinformatics* 2014, **30**(7):923-930.
11. Robinson MD, Oshlack A: **A scaling normalization method for differential expression analysis of RNA-seq data**. *Genome Biol* 2010, **11**(3):R25.
12. Plaisier SB, Taschereau R, Wong JA, Graeber TG: **Rank-rank hypergeometric overlap: identification of statistically significant overlap between gene-expression signatures**. *Nucleic Acids Res* 2010, **38**(17):e169.
13. Barabasi AL, Oltvai ZN: **Network biology: understanding the cell's functional organization**. *Nat Rev Genet* 2004, **5**(2):101-113.
14. Hric D, Darst RK, Fortunato S: **Community detection in networks: Structural communities versus ground truth**. *Phys Rev E Stat Nonlin Soft Matter Phys* 2014, **90**(6):062805.
15. Cowley GS, Weir BA, Vazquez F, Tamayo P, Scott JA, Rusin S, East-Seletsky A, Ali LD, Gerath WF, Pantel SE *et al*: **Parallel genome-scale loss of function screens in 216 cancer cell lines for the identification of context-specific genetic dependencies**. *Sci Data* 2014, **1**:140035.
16. Subramanian A, Tamayo P, Mootha VK, Mukherjee S, Ebert BL, Gillette MA, Paulovich A, Pomeroy SL, Golub TR, Lander ES *et al*: **Gene set enrichment analysis: a knowledge-based approach for interpreting genome-wide expression profiles**. *Proc Natl Acad Sci U S A* 2005, **102**(43):15545-15550.
17. Szklarczyk D, Franceschini A, Wyder S, Forslund K, Heller D, Huerta-Cepas J, Simonovic M, Roth A, Santos A, Tsafou KP *et al*: **STRING v10: protein-protein interaction networks, integrated over the tree of life**. *Nucleic Acids Res* 2015, **43**(Database issue):D447-452.
18. Xue Y, Li Y, Guo R, Ling C, Wang W: **FANCM of the Fanconi anemia core complex is required for both monoubiquitination and DNA repair**. *Hum Mol Genet* 2008, **17**(11):1641-1652.
19. Kroismayr R, Baranyi U, Stehlik C, Dorfleutner A, Binder BR, Lipp J: **HERC5, a HECT E3 ubiquitin ligase tightly regulated in LPS activated endothelial cells**. *J Cell Sci* 2004, **117**(Pt 20):4749-4756.

20. Kuo CL, Goldberg AL: **Ubiquitinated proteins promote the association of proteasomes with the deubiquitinating enzyme Usp14 and the ubiquitin ligase Ube3c**. *Proc Natl Acad Sci U S A* 2017, **114**(17):E3404-E3413.
21. Xie CM, Wei W, Sun Y: **Role of SKP1-CUL1-F-box-protein (SCF) E3 ubiquitin ligases in skin cancer**. *J Genet Genomics* 2013, **40**(3):97-106.
22. Enserink JM, Kolodner RD: **An overview of Cdk1-controlled targets and processes**. *Cell Div* 2010, **5**:11.
23. Samuels Y, Waldman T: **Oncogenic mutations of PIK3CA in human cancers**. *Curr Top Microbiol Immunol* 2010, **347**:21-41.
24. Wei L, Surma M, Shi S, Lambert-Cheatham N, Shi J: **Novel Insights into the Roles of Rho Kinase in Cancer**. *Arch Immunol Ther Exp (Warsz)* 2016, **64**(4):259-278.
25. Cowper AE, Caceres JF, Mayeda A, Screaton GR: **Serine-arginine (SR) protein-like factors that antagonize authentic SR proteins and regulate alternative splicing**. *J Biol Chem* 2001, **276**(52):48908-48914.
26. Shkreta L, Toutant J, Durand M, Manley JL, Chabot B: **SRSF10 Connects DNA Damage to the Alternative Splicing of Transcripts Encoding Apoptosis, Cell-Cycle Control, and DNA Repair Factors**. *Cell Rep* 2016, **17**(8):1990-2003.
27. Dardenne E, Polay Espinoza M, Fattet L, Germann S, Lambert MP, Neil H, Zonta E, Mortada H, Gratadou L, Deygas M *et al*: **RNA helicases DDX5 and DDX17 dynamically orchestrate transcription, miRNA, and splicing programs in cell differentiation**. *Cell Rep* 2014, **7**(6):1900-1913.
28. Podhorecka M, Skladanowski A, Bozko P: **H2AX Phosphorylation: Its Role in DNA Damage Response and Cancer Therapy**. *J Nucleic Acids* 2010, **2010**.
29. Ohbayashi T, Oikawa K, Yamada K, Nishida-Umehara C, Matsuda Y, Satoh H, Mukai H, Mukai K, Kuroda M: **Unscheduled overexpression of human WAPL promotes chromosomal instability**. *Biochem Biophys Res Commun* 2007, **356**(3):699-704.
30. Lei L, Bandola-Simon J, Roche PA: **Ubiquitin-conjugating enzyme E2 D1 (Ube2D1) mediates lysine-independent ubiquitination of the E3 ubiquitin ligase March-I**. *J Biol Chem* 2018, **293**(11):3904-3912.
31. Beaudenon SL, Huacani MR, Wang G, McDonnell DP, Huibregtse JM: **Rsp5 ubiquitin-protein ligase mediates DNA damage-induced degradation of the large subunit of RNA polymerase II in Saccharomyces cerevisiae**. *Mol Cell Biol* 1999, **19**(10):6972-6979.
32. Corkery DP, Holly AC, Lahsaee S, Dellaire G: **Connecting the speckles: Splicing kinases and their role in tumorigenesis and treatment response**. *Nucleus* 2015, **6**(4):279-288.
33. Loughlin FE, Mansfield RE, Vaz PM, McGrath AP, Setiyaputra S, Gamsjaeger R, Chen ES, Morris BJ, Guss JM, Mackay JP: **The zinc fingers of the SR-like protein ZRANB2 are single-stranded RNA-binding domains that recognize 5' splice site-like sequences**. *Proc Natl Acad Sci U S A* 2009, **106**(14):5581-5586.
34. Wang R, Cukerman E, Heng HH, Liew CC: **Identification of a locus of zinc finger genes in human chromosome 19q13.1-q13.3 region by fluorescence in situ hybridization**. *Somat Cell Mol Genet* 1996, **22**(3):245-248.
35. Marechal A, Zou L: **DNA damage sensing by the ATM and ATR kinases**. *Cold Spring Harb Perspect Biol* 2013, **5**(9).
36. Goodwin EC, Motamedi N, Lipovsky A, Fernandez-Busnadiego R, DiMaio D: **Expression of DNAJB12 or DNAJB14 causes coordinate invasion of the nucleus by membranes associated with a novel nuclear pore structure**. *PLoS One* 2014, **9**(4):e94322.
37. Dekker SL, Kampinga HH, Bergink S: **DNAJs: more than substrate delivery to HSPA**. *Front Mol Biosci* 2015, **2**:35.

38. Starokadomskyy P, Gemelli T, Rios JJ, Xing C, Wang RC, Li H, Pokatayev V, Dozmorov I, Khan S, Miyata N *et al*: **DNA polymerase-alpha regulates the activation of type I interferons through cytosolic RNA:DNA synthesis**. *Nat Immunol* 2016, **17**(5):495-504.
39. Kershnar E, Wu SY, Chiang CM: **Immunoaffinity purification and functional characterization of human transcription factor IIH and RNA polymerase II from clonal cell lines that conditionally express epitope-tagged subunits of the multiprotein complexes**. *J Biol Chem* 1998, **273**(51):34444-34453.
40. Yoshimoto R, Kataoka N, Okawa K, Ohno M: **Isolation and characterization of post-splicing lariat-intron complexes**. *Nucleic Acids Res* 2009, **37**(3):891-902.
41. Shao S, von der Malsburg K, Hegde RS: **Listerin-dependent nascent protein ubiquitination relies on ribosome subunit dissociation**. *Mol Cell* 2013, **50**(5):637-648.
42. Kwak KS, Zhou X, Solomon V, Baracos VE, Davis J, Bannon AW, Boyle WJ, Lacey DL, Han HQ: **Regulation of protein catabolism by muscle-specific and cytokine-inducible ubiquitin ligase E3alpha-II during cancer cachexia**. *Cancer Res* 2004, **64**(22):8193-8198.
43. Hakimi MA, Bochar DA, Schmiesing JA, Dong Y, Barak OG, Speicher DW, Yokomori K, Shiekhattar R: **A chromatin remodelling complex that loads cohesin onto human chromosomes**. *Nature* 2002, **418**(6901):994-998.
44. Wu MY, Eldin KW, Beaudet AL: **Identification of chromatin remodeling genes Arid4a and Arid4b as leukemia suppressor genes**. *J Natl Cancer Inst* 2008, **100**(17):1247-1259.
45. Dias Carvalho P, Guimaraes CF, Cardoso AP, Mendonca S, Costa AM, Oliveira MJ, Velho S: **KRAS Oncogenic Signaling Extends beyond Cancer Cells to Orchestrate the Microenvironment**. *Cancer Res* 2018, **78**(1):7-14.
46. Chien AJ, Moore EC, Lonsdorf AS, Kulikauskas RM, Rothberg BG, Berger AJ, Major MB, Hwang ST, Rimm DL, Moon RT: **Activated Wnt/beta-catenin signaling in melanoma is associated with decreased proliferation in patient tumors and a murine melanoma model**. *Proc Natl Acad Sci U S A* 2009, **106**(4):1193-1198.
47. Hanahan D, Weinberg RA: **Hallmarks of cancer: the next generation**. *Cell* 2011, **144**(5):646-674.
48. Meehan WJ, Samant RS, Hopper JE, Carrozza MJ, Shevde LA, Workman JL, Eckert KA, Verderame MF, Welch DR: **Breast cancer metastasis suppressor 1 (BRMS1) forms complexes with retinoblastoma-binding protein 1 (RBP1) and the mSin3 histone deacetylase complex and represses transcription**. *J Biol Chem* 2004, **279**(2):1562-1569.
49. Nakayama K, Nakayama N, Wang TL, Shih Ie M: **NAC-1 controls cell growth and survival by repressing transcription of Gadd45GIP1, a candidate tumor suppressor**. *Cancer Res* 2007, **67**(17):8058-8064.
50. Cramer JM, Scarsdale JN, Walavalkar NM, Buchwald WA, Ginder GD, Williams DC, Jr.: **Probing the dynamic distribution of bound states for methylcytosine-binding domains on DNA**. *J Biol Chem* 2014, **289**(3):1294-1302.
51. Euskirchen G, Auerbach RK, Snyder M: **SWI/SNF chromatin-remodeling factors: multiscale analyses and diverse functions**. *J Biol Chem* 2012, **287**(37):30897-30905.
52. Xu H, Washington S, Verderame MF, Manni A: **Activation of protein kinase A (PKA) signaling mitigates the antiproliferative and antiinvasive effects of alpha-difluoromethylornithine in breast cancer cells**. *Breast Cancer Res Treat* 2008, **107**(1):63-70.
53. Mayeda A, Badolato J, Kobayashi R, Zhang MQ, Gardiner EM, Krainer AR: **Purification and characterization of human RNPS1: a general activator of pre-mRNA splicing**. *EMBO J* 1999, **18**(16):4560-4570.
54. Kim YS, Kang KR, Wolff EC, Bell JK, McPhie P, Park MH: **Deoxyhypusine hydroxylase is a Fe(II)-dependent, HEAT-repeat enzyme. Identification of amino acid residues critical for Fe(II) binding and catalysis [corrected]**. *J Biol Chem* 2006, **281**(19):13217-13225.

55. Roszer T: **Understanding the Mysterious M2 Macrophage through Activation Markers and Effector Mechanisms**. *Mediators Inflamm* 2015, **2015**:816460.
