# Supplementary material for: Network models of primary melanoma microenvironments identify key melanoma regulators underlying prognosis
Source: Nat Commun. 2021 Feb 22;12:1214. doi: 10.1038/s41467-021-21457-0 (PMC7900178; doi:10.1038/s41467-021-21457-0)
Supplement: Supplementary file 11 — Reporting Summary [file 41467_2021_21457_MOESM11_ESM.pdf]

nature research

Corresponding author(s): Eva Hernando, Bin Zhang

Last updated by author(s): Dec 22, 2020

Reporting Summary

Nature Research wishes to improve the reproducibility of the work that we publish. This form provides structure for consistency and transparency in reporting. For further information on Nature Research policies, see our Editorial Policies and the Editorial Policy Checklist.

Statistics

For all statistical analyses, confirm that the following items are present in the figure legend, table legend, main text, or Methods section.

n/a	Confirmed	
☐	☒	The exact sample size (n) for each experimental group/condition, given as a discrete number and unit of measurement
☐	☒	A statement on whether measurements were taken from distinct samples or whether the same sample was measured repeatedly
☐	☒	The statistical test(s) used AND whether they are one- or two-sided<br>*Only common tests should be described solely by name; describe more complex techniques in the Methods section.*
☐	☒	A description of all covariates tested
☐	☒	A description of any assumptions or corrections, such as tests of normality and adjustment for multiple comparisons
☐	☒	A full description of the statistical parameters including central tendency (e.g. means) or other basic estimates (e.g. regression coefficient) AND variation (e.g. standard deviation) or associated estimates of uncertainty (e.g. confidence intervals)
☐	☒	For null hypothesis testing, the test statistic (e.g. F, t, r) with confidence intervals, effect sizes, degrees of freedom and P value noted<br>*Give P values as exact values whenever suitable.*
☒	☐	For Bayesian analysis, information on the choice of priors and Markov chain Monte Carlo settings
☐	☒	For hierarchical and complex designs, identification of the appropriate level for tests and full reporting of outcomes
☐	☒	Estimates of effect sizes (e.g. Cohen's d, Pearson's r), indicating how they were calculated

Our web collection on statistics for biologists contains articles on many of the points above.

Software and code

Policy information about availability of computer code

Data collection	MSigDB (v5.0), GSE72056 (from GEO, single cell transcriptome), Jerby-Arnon et al 2018 (from published data, see manuscript for reference), TCGA SKCM (subsetted for primary samples with sample code 01, bulk transcriptome), Van Allen et al 2015 (from published data, see manuscript for reference)
Data analysis	R (v3.6.0), MEGENA (v1.4), CIBERSORT (v1.04), estimate (v1.0.13), SingleCellExperiment (v1.10.0), scater (v1.16.0), scran (v1.16.0), ggplot2 (v3.3.2)

For manuscripts utilizing custom algorithms or software that are central to the research but not yet described in published literature, software must be made available to editors and reviewers. We strongly encourage code deposition in a community repository (e.g. GitHub). See the Nature Research guidelines for submitting code & software for further information.

Data

Policy information about availability of data

All manuscripts must include a data availability statement. This statement should provide the following information, where applicable:

- Accession codes, unique identifiers, or web links for publicly available datasets
- A list of figures that have associated raw data
- A description of any restrictions on data availability

The original gene expression data, analyzed as primary melanoma (pSKCM), are available from The Cancer Genome Atlas (TCGA) data portal (https://portal.gdc.cancer.gov/). The single cell RNA sequencing data is available from Gene Expression Omnubus (GEO) under accession number GSE72056, and we downloaded the normalized TPM values (file name: GSE72056_melanoma_single_cell_revised_v2.txt.gz). Additionally, the processed data are available from the corresponding author upon reasonable request.

Field-specific reporting

Please select the one below that is the best fit for your research. If you are not sure, read the appropriate sections before making your selection.

- [x] Life sciences
- [ ] Behavioural & social sciences
- [ ] Ecological, evolutionary & environmental sciences

For a reference copy of the document with all sections, see nature.com/documents/nr-reporting-summary-flat.pdf

Life sciences study design

All studies must disclose on these points even when the disclosure is negative.

Sample size	Computational study: The main discovery cohort of primary melanoma samples from TCGA (pSKCM) is composed of 103 samples. For validation bulk cohorts, the metastatic samples from TCGA (mSKCM) has 353 samples, and Van Allen et al. 2015 cohort contains 40 samples. The discovery single cell transcriptome (GSE72056) contains 4645 cells. The validation single-cell transcriptome data from Jerby-Arnon et al. 2018 contains 36,744 cells.<br>Experimental Study: For in vivo studies, 12 mice (NSG) per group were used as detailed in experimental procedures.
Data exclusions	N/A
Replication	Computational Study: To test reproducibility of ZNF180-centered network models, we applied the same co-expression network algorithm (i.e. MEGENA) from the discovery bulk cohort (pSKCM) to the independent validation cohorts (mSKCM, Van Allen et al. 2015), then tested enrichments of ZNF180 knock-down signatures in their respective ZNF180 neighborhoods. To test reproducibility of MYO1F-driven macrophage M1-polarization, the same analytical steps in the discovery single-cell data (i.e. GSE72056) were applied to the independent single-cell data (Jerby-Arnon et al. 2018) to establish MYO1F expressions in M1-macrophages. The correlation between MYO1F expression and M1-macrophage abundance was also replicated in the validation bulk cohort (Van Allen et al. 2015) by repeating CIBERSORT analysis as applied in the discovery bulk cohort (pSKCM).<br>Experimental Study: For in vitro functional assays such as Transwell matrigel invasion and cell proliferation results were presented as mean +/- standard deviation of scale normalized, composite data from three and two biological replicate experiments respectively. Statistical analyses were performed by two-tailed Student's t test. RT-qPCR analyses was performed in biological triplicates and analyzed by the delta delta Ct method using normalization to GAPDH gene. For in vivo studies, 12 mice (NSG, Jackson labs #005557) per group were used as detailed in experimental procedures. Statistical significance between groups were analyzed using two-tailed Student's t test.
Randomization	N/A
Blinding	N/A

Reporting for specific materials, systems and methods

We require information from authors about some types of materials, experimental systems and methods used in many studies. Here, indicate whether each material, system or method listed is relevant to your study. If you are not sure if a list item applies to your research, read the appropriate section before selecting a response.

Materials & experimental systems

n/a	Involved in the study	
x		Antibodies
	x	Eukaryotic cell lines
x		Palaeontology and archaeology
	x	Animals and other organisms
x		Human research participants
x		Clinical data
x		Dual use research of concern

Methods

n/a	Involved in the study	
x		ChIP-seq
x		Flow cytometry
x		MRI-based neuroimaging

Eukaryotic cell lines

Policy information about cell lines

Cell line source(s)	SKmel147 was obtained from Dr. Alan Houghton's laboratory (MSKCC, New York). A375 was purchased from American Type Culture Collection (ATCC).
Authentication	Melanoma cell lines SkMel147 and A375 were authenticated by Short Tandem Repeat (STR) analysis by ATCC.
Mycoplasma contamination	Cell lines were routinely tested to exclude Mycoplasma contamination.
Commonly misidentified lines (See ICLAC register)	*Name any commonly misidentified cell lines used in the study and provide a rationale for their use.*

Animals and other organisms

Policy information about studies involving animals; ARRIVE guidelines recommended for reporting animal research

Laboratory animals	NOD/Shi-scid/IL-2Rgamma null (NSG, Jackson labs #005557) 20-weeks-old male mice (n=12 per group)
Wild animals	N/A
Field-collected samples	N/A
Ethics oversight	All mice experiments were performed in compliance with a referenced protocol # (S16-00051) approved by the NYU institutional animal care and use committee (IACUC).

Note that full information on the approval of the study protocol must also be provided in the manuscript.
