# Supplementary material for: Network models of primary melanoma microenvironments identify key melanoma regulators underlying prognosis
Source: Nat Commun. 2021 Feb 22;12:1214. doi: 10.1038/s41467-021-21457-0 (PMC7900178; doi:10.1038/s41467-021-21457-0)
Supplement: Supplementary file 12 — Description of Additional Supplementary Files [file 41467_2021_21457_MOESM12_ESM.pdf]

Description of Additional Supplementary Files

Supplementary Information. Supplementary Notes, Tables and Figures.

Supplementary Data 1. Prognostic gene signatures from primary and metastatic tumors of TCGA melanoma samples. **A**. List of overall survival signature with logrank p-value < 0.05 within primary (primary good, primary poor), metastatic (metastatic good, metastatic poor) or common to both (common good, common poor, common signature). **B**. Survival signature from A in .GMT format.

Supplementary Data 2. A. Ranked list of 221 modules by enrichment prognostic gene signatures in both of primary and metastatic tumors. **B.** Refined list of 84 modules by screening for parent-child relationship and differential connectivity compared to normal skin data from GTEx.

Supplementary Data 3. List of significantly enriched MSigDB signatures for hub genes of module M7 with Bonferroni corrected FET p-value < 0.05.

Supplementary Data 4. **A**. Cell information of GSE72056 scRNA-seq data. **B**. Cell information of Jerby-Arnon *et al*. 2018 scRNA-seq data. **C**. Enrichments of bulk-based pSKCM modules in cell type-specific network modules from scRNA-seq with FDR corrected FET p-value < 0.05.

Supplementary Data 5. List of significant correlations between methylation profiles and gene expressions for pSKCM cohort with Bonferroni corrected Spearman correlation p-value < 0.05.

Supplementary Data 6. List of significant causal trios anchored by methylations is identified by Causality Inference Test (CIT) framework with CIT p-value < 0.05.

Supplementary Data 7. Inferred pSKCM cell populations from **A**. CIBERSORT, and **B**. ESTIMATE. **C**. Inferred cell types from GSE72056 scRNA-seq by decision-tree based algorithm. **D**. CPM inferred GSE72056 cell compositions in pSKCM. **E**. Cell types enriched for cells expressing individual genes from top 10 modules. The enrichments were evaluated by Fisher's Exact Test, and p-values were corrected by Benjamini-Hochberg False Discovery Rate (BH FDR).

Supplementary Data 8. A. Differentially expressed genes in siZNF180/siZNF347/siPPP1R2 compared to non-transfected control (NTC). **B**. Gene expressions from siRNA transfected SKmel147 cells (siZNF347, siPPP1R2, siZNF180) and non-transfected controls (siNTC).

Supplementary Data 9. Significant correlations between gene expressions and RPPA protein expressions in pSKCM with FDR < 0.05.
