# Supplementary material for: Network models of primary melanoma microenvironments identify key melanoma regulators underlying prognosis
Source: Nat Commun. 2021 Feb 22;12:1214. doi: 10.1038/s41467-021-21457-0 (PMC7900178; doi:10.1038/s41467-021-21457-0)
Supplement: Supplementary file 13 — Source Data [file 41467_2021_21457_MOESM13_ESM.zip › 220894_2_related_ms_5203725_qm38tf (1).pptx]

## Slide 1
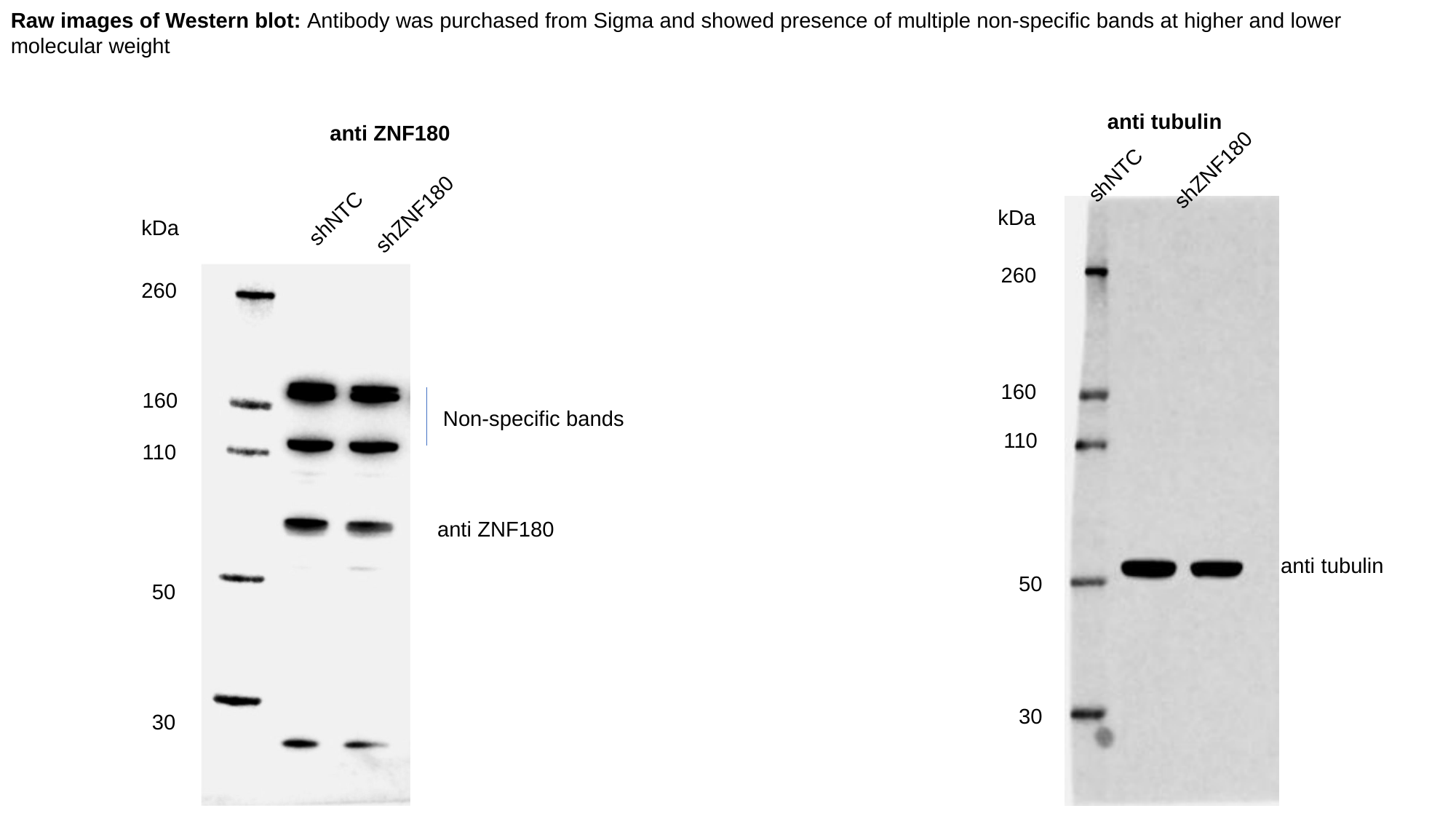

Raw images of Western blot: Antibody was purchased from Sigma and showed presence of multiple non-specific bands at higher and lower molecular weight
anti tubulin
kDa
260
160
110
anti tubulin
50
30
anti ZNF180
kDa
260
160
Non-specific bands
110
anti ZNF180
50
30
shZNF180
shNTC
shNTC
shZNF180
